# Toxicokinetic Modeling of Persistent Organic Pollutant Levels in Blood from Birth to 45 Months of Age in Longitudinal Birth Cohort Studies

**DOI:** 10.1289/ehp.1205552

**Published:** 2012-10-17

**Authors:** Marc-André Verner, Dean Sonneborn, Kinga Lancz, Gina Muckle, Pierre Ayotte, Éric Dewailly, Anton Kocan, Lubica Palkovicová, Tomas Trnovec, Sami Haddad, Irva Hertz-Picciotto, Merete Eggesbø

**Affiliations:** 1 Institute of Environmental Medicine, Karolinska Institutet, Solna, Sweden; 2 Channing Division of Network Medicine, Brigham and Women’s Hospital, Harvard Medical School, Boston, Massachusetts, USA; 3 Department of Public Health Sciences, University of California, Davis, Davis, California, USA; 4 Department of Environmental Medicine, Slovak Medical University, Bratislava, Slovakia; 5 Centre de recherche du CHUQ-CHUL, Université Laval, Quebec City, Quebec, Canada; 6 Research Centre for Toxic Compounds in the Environment, Faculty of Science, Masaryk University, Brno, Czech Republic; 7 Department of Environmental Health and Occupational Health, IRSPUM (Université de Montréal Public Health Research Institute), Université de Montréal, Montreal, Quebec, Canada; 8 Department of Genes and Environment, Norwegian Institute of Public Health, Oslo, Norway

**Keywords:** children’s health, lactational exposures, longitudinal birth studies, persistent organic pollutants, toxicokinetic modeling

## Abstract

Background: Despite experimental evidence that lactational exposure to persistent organic pollutants (POPs) can impact health, results from epidemiologic studies are inconclusive. Inconsistency across studies may reflect the inability of current methods to estimate children’s blood levels during specific periods of susceptibility.

Objectives: We developed a toxicokinetic model to simulate blood POP levels in children from two longitudinal birth cohorts and aimed to validate it against blood levels measured at 6, 16, and 45 months of age.

Methods: The model consisted of a maternal and a child lipid compartment connected through placental diffusion and breastfeeding. Simulations were carried out based on individual physiologic parameters; duration of breastfeeding; and levels of POPs measured in maternal blood at delivery, cord blood, or breast milk. Model validity was assessed through regression analyses of simulated against measured blood levels.

Results: Simulated levels explained between 10% and 83% of measured blood levels depending on the cohort, the compound, the sample used to simulate children’s blood levels, and child’s age when blood levels were measured. Model accuracy was highest for estimated blood POP levels at 6 months based on maternal or cord blood levels. However, loss in model precision between the 6th and the 45th month was small for most compounds.

Conclusions: Our validated toxicokinetic model can be used to estimate children’s blood POP levels in early to mid-childhood. Estimates can be used in epidemiologic studies to evaluate the impact of exposure during hypothesized postnatal periods of susceptibility on health.

Many persistent organic pollutants (POPs) can be measured in human breast milk samples collected worldwide. Early exposure to POPs through breastfeeding significantly increases children’s body burden (Lanting et al. 1998; Patandin et al. 1997; Ribas-Fito et al. 2005) and is thought to be the primary determinant of children’s blood levels until at least 7 years of age (Barr et al. 2006). The impact of postnatal intake of POPs on child development has been assessed in epidemiologic studies but results are mostly inconsistent (Jorissen 2007), possibly because of limitations in current methods used to assess exposure.

A handful of approaches were proposed to characterize children’s exposure to POPs via breastfeeding in epidemiologic studies. Huisman et al. (1995) used the concentration of polychlorinated biphenyls (PCBs) and dioxins in breast milk lipids as an estimate of postnatal exposure. To account for duration of lactation, several epidemiologic studies multiplied breast milk concentration by the duration of breastfeeding (Grandjean et al. 2003; Jacobson and Jacobson 1996; Koopman-Esseboom et al. 1996; Rogan et al. 1987). Pan et al. (2009) developed a similar exposure metric, but they distinguished exclusive from partial breastfeeding periods, and assumed that breast milk consumption when children were also given other supplements is half that of children being exclusively breastfed. Direct measurement of POPs in children’s blood lipids was also used as a proxy of postnatal internal exposure (Boucher et al. 2012; Grandjean et al. 2003; Jacobson and Jacobson 1996; Plusquellec et al. 2010; Saint-Amour et al. 2006). None of these methods generates complete profiles of tissue or blood levels across development. Overall estimates may fail to detect associations with POPs that affect health during narrow time windows of exposure only (Hertz-Picciotto et al. 1996).

In a previous longitudinal birth cohort study, we estimated children’s blood POP profile over the first year of life with a validated physiologically based pharmacokinetic (PBPK) model (Verner et al. 2010). Generating profiles of postnatal blood PCB-153 levels allowed us to assess exposure during different windows, an approach that led us to identify a postnatal interval of susceptibility to neurotoxic insults. The PBPK model was, however, quite complex and did not allow the incorporation of certain individual variables such as gestational age, weight gain during pregnancy, and weight loss after delivery, all of which may modulate children’s exposure. Furthermore, this model required information on mothers’ and children’s height at different times, which may not be systematically documented in epidemiologic studies. Finally, the model was validated only for simulations based on maternal blood levels and only until 6 months of age.

To overcome these limitations and ease the use of toxicokinetic modeling in epidemiologic studies, we developed a simplified model to simulate blood POP levels across childhood and validated it to 45 months of age using blood levels of children enrolled in two longitudinal birth cohorts. We also assessed model validity for simulations based on levels in different samples: maternal blood, cord blood, and breast milk. We confined our analyses to four PCB congeners [International Union of Pure and Applied Chemistry (IUPAC) no. 118, 153, 170, and 180], 1,1-dichloro-2,2-bis(*p*-chlorophenyl)ethylene (*p,p´*-DDE), 1,1,1-trichloro-2,2-bis(*p*-chlorophenyl)ethane (*p,p´*-DDT) and hexachlorobenzene (HCB).

## Methods

**Figure 1:**
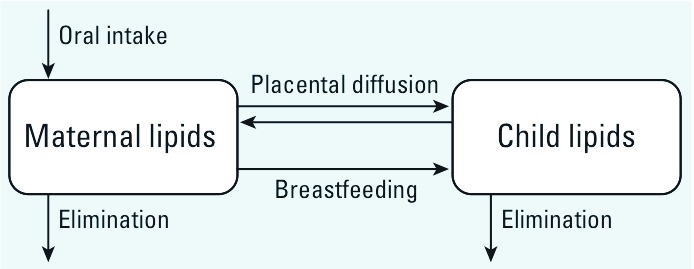
Conceptual representation of the toxicokinetic model.

*Conceptual representation.* We developed this model based on the assumption that POPs distribute almost exclusively in lipids (Emond et al. 2005; Haddad et al. 2000). Therefore, we represented both the mother and child as lipid compartments (including blood lipids) connected through placental diffusion and excretion/intake of breast milk (Figure 1). The mother was exposed to POPs through ingestion of contaminated food. The child was exposed prenatally through placental diffusion and postnatally through consumption of breast milk.

*Parameterization.* The complete set of parameters and equations is available in the model code provided in Supplemental Material (http://dx.doi.org/10.1289/ehp.1205552). Briefly, the kinetics of POPs are described as follows:

Absorption. POPs in the gastrointestinal tract of both mothers and children were assumed to be fully absorbed as suggested by results from experimental studies (McLachlan 1993).

Distribution. Following absorption, POPs were assumed to distribute homogeneously in body lipids. During pregnancy, POPs were allowed to partition equally in maternal and fetal lipids. Mothers’ and children’s lipid volumes were scaled to individual-specific age and body weight according to data from Fomon et al. (1982) and the International Commission on Radiological Protection (ICRP 2002). The percentage of lipids in fetal tissues across pregnancy was set to values at birth—15% for males and 14% for females. Gain in body fat mass during pregnancy was calculated as the difference between weight gain and increase in lean tissues (fetus, uterus, placenta, amniotic fluid, blood, breasts, and extracellular and extravascular fluid). Fetal growth was based on a general growth curve (ICRP 2002) and individual birth weight, whereas increase in other lean tissues was the same for all women (increase of 5.8 kg at the end of pregnancy) (ICRP 2002). After delivery, the difference between weight and prepregnancy weight was considered to be strictly attributable to adipose tissue. This assumption was based on a study reporting that lean tissue gain during pregnancy is rapidly lost after delivery (Butte et al. 2003). The additional adipose tissue during and after pregnancy was assumed to be composed of 75% lipids (Baker 1969).

Metabolism and excretion. Elimination due to biotransformation and fecal excretion was set as an output from mother and children compartments with rates based on half-lives derived from cross-sectional data [PCB-118: 9.3 years; PCB-138: 10.8 years; PCB-153: 14.4 years; PCB-170: 15.5 years; PCB-180: 11.5 years (Ritter et al. 2011); *p,p´-*DDT: ~ 5 years (Smith 1999)] and longitudinal data [*p,p´*-DDE: 13 years (Wolff et al. 2000); HCB: 6 years (To-Figueras et al. 2000)]. Elimination rates (*K*_el_) were calculated as follows:

*K*_el_ = ln(2)/half-life. [1]

Excretion of POPs in breast milk lipids was driven by children’s breast milk consumption and breast milk lipid content. Milk consumption during exclusive breastfeeding was described based on data from Salmenpera et al. (1985), expressed on the basis of children’s body weight and age by Arcus-Arth et al. (2005):

Hourly milk intake (L/kg body weight)  = –0.0024 × Age (years) + 0.0063. [2]

From 12 until 24 months of age, we described breast milk consumption based on partial breastfeeding data published by Kent et al. (1999):

Hourly milk intake (L)  = –0.0086 × Age (years) + 0.0188. [3]

We described breast milk lipids based on concentrations published by Bitman et al. (1983) and Arcus-Arth et al. (2005):

Fraction of lipids in breast milk (kg/L) = 0.0034 × ln(Age [years]) + 0.0414. [4]

*Model inputs.* To generate postnatal exposure profiles, the model requires the inclusion of certain variables that are relevant to POP toxicokinetics: age of mother at delivery, prepregnancy body weight, child’s birth weight, child’s weight at one or more postnatal times (along with the time of measurement), duration of exclusive breastfeeding, duration of partial breastfeeding (when the child is fed both breast milk and other food), child’s sex, levels of POPs in maternal blood, cord blood or breast milk lipids (with the time of sampling), and half-life of the POP to be modeled. In addition to these essential variables, the model can incorporate data on: weight gain during pregnancy and weight changes after delivery, gestational age at birth, and percentage of food intake attributable to breast milk during partial breastfeeding.

*Inuit birth cohort.* Pregnant Inuit women from Northern Quebec (Canada) were invited to participate in this longitudinal birth cohort shortly after their first prenatal visit (Muckle et al. 2001). Maternal and cord blood samples were collected at the time of delivery. Approximately 1 month after delivery, a sample of breast milk was obtained from mothers. Children’s blood was drawn around 6 months (range, 1–14 months) after birth. A large array of organochlorines including pesticides and PCBs was analyzed in these samples, as described by Muckle et al. (2001). In this cohort, information on breastfeeding was collected 6 and 11 months after delivery. Only mother–child dyads with POP levels above the limit of detection and available data on duration of exclusive and partial breastfeeding were included in the validation analyses. Missing maternal and child values for other variables were replaced by the variable’s mean value. A total of 160 children were included in the analyses. Detailed informed consent was provided by each participating mother. The research procedures were approved by the human subjects committees of Laval University and Wayne State University.

*Slovak birth cohort.* Women from the regions of Michalovce and Svidnik/Stropkov in Slovakia were invited to enroll in this longitudinal birth cohort at the time of delivery (Hertz-Picciotto et al. 2003). Maternal and cord blood samples were collected at the time of delivery. Blood samples were drawn from children at 6 months (range, 5.5–11.7), 16 months (range, 11.8–22.0), and 45 months of age (range, 33.0–69.1). Mothers enrolled in this study were asked about their breastfeeding practices 6 months and 16 months after delivery. Available data on the duration of exclusive and partial breastfeeding and PCB levels above the limit of detection were the only inclusion criteria for validation analyses. A total of 795 children were included in these analyses. This study was approved by Institutional Review Boards of the University of California, Davis (USA) and the Slovak Medical University in Bratislava, Slovakia. Written informed consent was provided by all participants, and consent was given by the parents for infants to participate in the study.

**Figure 2:**
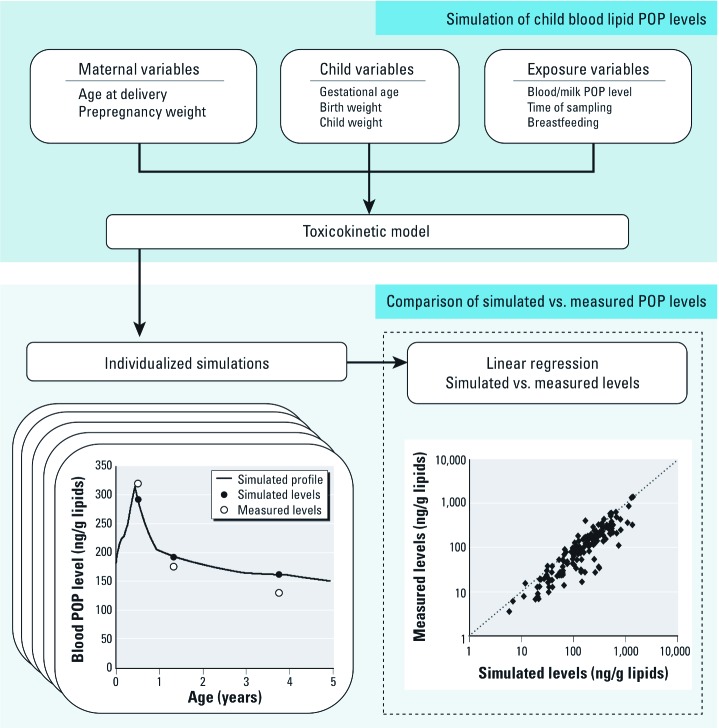
Steps undertaken to validate the toxicokinetic model.

*Validation.* We validated the model following the steps presented in Figure 2. For both cohorts, individual-specific variables on age at delivery, prepregnancy weight, child weight, duration of exclusive and partial breastfeeding, and child sex were incorporated in the toxicokinetic model. Average values were used for weight gain during pregnancy (14.5 kg; Butte et al. 2003) and weight changes postpartum (2 kg above prepregnancy weight 0.5 year after delivery; Butte et al. 2003) because individual data were not collected in these cohorts. In addition, we assumed that maternal weight returned to prepregnancy weight by the end of the first year, and that breast milk consumption during periods of partial breastfeeding was 50% of the amount consumed by exclusively breastfed children at the same age.

We first estimated maternal lifetime daily POP oral intake based on levels measured in maternal blood at delivery, cord blood, or breast milk. We subsequently performed simulations of children’s POP levels for each of the available samples (maternal blood, cord blood, and/or breast milk) and compared simulated with measured POP levels by linear regression to determine how much of the variability in measured blood levels could be explained by the toxicokinetic model based on the coefficient of determination (*R*^2^). To avoid bias related to heteroskedasticity, measured and simulated levels were log-transformed before regression analyses.

*Global sensitivity analysis.* To quantify the impact of the various model inputs on simulated children’s blood POP levels, we ran a sensitivity analysis where model input values were iteratively sampled from specified ranges: prepregnancy body weight (51–114 kg; McDowell et al. 2005), weight gain during pregnancy (7.6–32.0 kg; Sohlstrom and Forsum 1995), postpartum weight changes (–12 to 26 kg 1 year after delivery; Rossner 1997), gestational age (22–42 weeks; Kramer et al. 2001), birth weight (0.34–4.9 kg; Kramer et al. 2001), weight at 6 months (6.4–9.8 kg; Kuczmarski et al. 2000), weight at 1 year (8.4–12.8 kg; Kuczmarski et al. 2000), weight at 3 years (11.8–18 kg; Kuczmarski et al. 2000), duration of exclusive breastfeeding (0–12 months; arbitrary), duration of partial breastfeeding (0–45 months; arbitrary), fraction of food intake attributable to breast milk during partial breastfeeding (0.1–0.9; arbitrary, single percentage used for the whole period of partial breastfeeding).

In the model, certain parameters are likely to interact with each other (e.g., duration of partial breastfeeding and fraction of food intake attributable to breast milk), and the sensitivities reported should ideally account for these interactions as thoroughly as possible. Commonly employed one-at-a-time local sensitivity analyses (where only one parameter is varied while the others are kept constant) cannot account for these interactive effects. For this reason, we opted for an extended Fourier amplitude sensitivity analysis (eFAST) (McNally et al. 2011). Although more computationally intensive, this global approach allows the quantitative calculation of the relative contributions for a set of parameters over ranges of physiologically appropriate values—a clear advantage over local sensitivity analyses. Global sensitivity (total effects) obtained for each parameter at different time points (6, 16, and 45 months) was used to indicate its relative influence on the model output. We ran separate sensitivity analyses for PCB-153, a compound with a relatively long half-life, and *p,p´-*DDT, a compound with a relatively short half-life, in boys and in girls.

*Exposure misclassification in epidemiologic studies.* To illustrate that a commonly employed method to estimate postnatal exposure can lead to exposure misclassification, we simulated profiles of blood PCB-153 levels in two Inuit children who had the same overall lactational exposure when estimated based only on the duration of breastfeeding and the concentration in breast milk (weeks breastfeeding × ng/g milk lipids = ~ 4,700).

*Model and software.* Toxicokinetic modeling was performed using acslX (Aegis Technologies Group, Inc., Huntsville, AL, USA) and Microsoft Excel® 2007 (Microsoft, Redmond, WA, USA). Statistical analyses were conducted using SPSS for Windows statistical package version 20 (IBM, Chicago, IL, USA). The model code and automation script are provided in the Supplemental Material (http://dx.doi.org/10.1289/ehp.1205552).

## Results

Inuit mothers who ever breastfed their child (89%) tended to do so exclusively for a longer period than their Slovak counterparts (100%). Moreover, the duration of exclusive breastfeeding was more variable in the Inuit cohort (Table 1). Levels of PCBs and other organochlorines in maternal and cord blood lipids were higher in the Slovak cohort, except for PCB-118, but the variability in levels, as indicated by 5th–95th percentile ranges and their ratios, was relatively comparable (Table 2).

**Table 1:** Demographic characteristics [mean (5th–95th percentile)].

Characteristic	Inuits (n = 160)	Slovaks (n = 795)
Age at delivery (years)	25 (17–36)	26 (19–34)
Prepregnancy weight (kg)	71.2 (55.3–93.7)	60.2 (46.0–81.0)
Gestational age at birth (weeks)	39 (36–41)	40 (38–41)
Birth weight (kg)	3.5 (2.4–4.4)	3.4 (2.6–4.2)
Child weight at 6 months (kg)	9.6 (7.8–11.5)	7.9 (6.2–9.6)
Child weight at 16 months (kg)	—	11.7 (9.0–14.3)
Child weight at 45 months (kg)	—	17.0 (13.0–22.6)
Exclusive breastfeeding (days)	185 (6–370)	96 (30–183)
Total breastfeeding (days)	220 (7–391)	205 (30–487)

**Table 2:** Median POP levels (ng/g lipids) in maternal blood at delivery, cord blood, and breast milk samples collected in the Inuit and Slovak studies.

Compound	Maternal blood			Cord blood			Breast milk
	Inuit (n = 156)	Slovak (n = 754)	Inuit (n = 81)	Slovak (n = 752)	Inuit (n = 130)
PCB-118	14 (5–46)	8 (1–45)	12 (4–57)	4 (1–38)	18 (6–71)
PCB-138	59 (21–167)	90 (32–360)	51 (16–200)	77 (21–345)	70 (22–262)
PCB-153	104 (32–336)	140 (53–553)	78 (22–319)	111 (34–457)	118 (41–412)
PCB-170	17 (6–65)	54 (20–209)	12 (4–51)	36 (8–154)	17 (4–75)
PCB-180	44 (14–155)	127 (49–523)	28 (9–122)	89 (25–376)	44 (15–167)
p,p´-DDE	281 (107–1,023)	432 (108–1,430)	276 (98–1,060)	409 (91–1,480)	384 (132–1,501)
p,p´-DDT	14 (3–38)	21 (7–82)	14 (5–40)	17 (4–65)	30 (8–95)
HCB	40 (15–122)	65 (5–302)	40 (17–148)	73 (5–329)	48 (15–133)
Values in parentheses represent the 5th and 95th percentiles.										

*Accuracy of simulations for different compounds.* There was little variation in model accuracy across PCB congeners in both cohorts for a given sample type and age, although *R*^2^ of measured against simulated levels were consistently higher in the Inuit cohort than in the Slovak cohort (Table 3). In the Slovak cohort, the explained variability was somewhat lower for *p,p´-*DDE and HCB than for PCBs. Model accuracy was lower for *p,p´-*DDT than for other compounds in the Inuit cohort.

**Table 3:** Regression analyses of blood POP levels measured at 6, 16, or 45 months of age against levels simulated from measured values in cord blood, maternal blood (at delivery), or breast milk (collected approximately 1 month postpartum).

Compound	Inuit cohort (months)					Slovak cohort (months)										
	Cord blood	Maternal blood	Breast milk	Cord blood					Maternal blood				
	6	6	6	6	16	45	6	16	45
PCB-118
R2	0.74	0.73	0.63	0.52	0.46	0.33	0.55	0.41	0.33
n	60	126	112	205	726	404	215	728	390
PCB-138
R2	0.75	0.80	0.63	0.59	0.49	0.49	0.60	0.54	0.51
n	79	147	118	205	726	405	216	728	391
PCB-153
R2	0.74	0.81	0.65	0.57	0.51	0.50	0.59	0.53	0.51
N	79	150	118	205	726	405	216	728	391
PCB-170
R2	0.72	0.65	0.66	0.50	0.46	0.45	0.56	0.53	0.50
n	51	111	103	205	726	405	216	728	391
PCB-180
R2	0.75	0.77	0.63	0.59	0.51	0.50	0.60	0.54	0.51
n	78	143	117	205	726	405	216	728	391
p,p´-DDE
R2	0.76	0.83	0.62	0.40	0.48	0.39	0.49	0.44	0.39
n	81	156	118	205	726	405	216	728	391
p,p´-DDT
R2	0.52	0.47	0.49	0.57	0.44	0.31	0.58	0.45	0.30
n	48	103	102	205	726	406	216	717	391
HCB
R2	0.63	0.74	0.59	0.44	0.40	0.13	0.40	0.37	0.10
n	66	144	116	198	718	395	210	723	383
R2 values represent the fraction of variability in measured levels explained by the toxicokinetic model.																		

**Figure 3:**
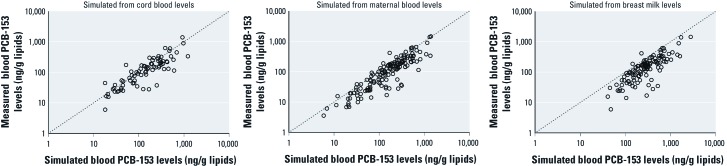
PCB-153 levels measured in children’s blood in the Inuit cohort plotted against blood PCB-153 levels simulated from cord blood, maternal blood at delivery, or breast milk concentrations. Axes are on a logarithmic scale.

*Sample used to simulate children’s blood levels.* In the Slovak cohort, the model had similar predictiveness at each age regardless of the type of sample (cord blood or maternal blood at delivery) used to simulate children’s blood levels (Table 3). Predictiveness was also similar between cord blood and maternal blood for the Inuit cohort, but accuracy was lower for simulations based on milk samples (Table 3) and tended to overestimate children’s blood levels by an average of 60% (Figure 3).

**Figure 4:**
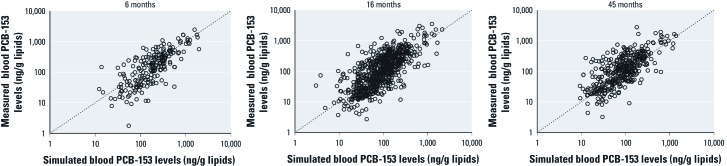
PCB-153 levels measured in children’s blood at 6, 16, and 45 months of age in the Slovak cohort plotted against blood PCB-153 levels simulated from cord blood concentrations. Axes are on a logarithmic scale.

*Validity across development.* Although the predictiveness of simulations for the Slovak cohort decreased as the age of children at blood sample measurement increased, the loss in accuracy was small for most compounds (Table 3, Figure 4). However, the decrease in *R*^2^ values was more pronounced for PCB-118, *p,p´-*DDT, and HCB, the compounds with the shortest half-lives.

**Figure 5:**
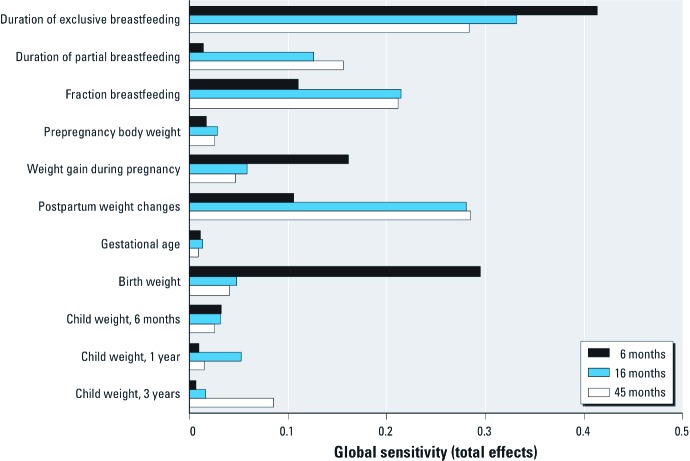
Global sensitivity analysis (eFAST) of the influence of model inputs on estimates of blood PCB-153 levels in girls at 6, 16, and 45 months of age. Results of global sensitivity analyses were comparable for *p,p´*-DDT and according to sex.

*Global sensitivity analysis.* The influence of model inputs on children’s blood POP levels was assessed using eFAST global sensitivity analysis. The global sensitivity (total effects) of the different parameters on POP levels varied depending on children’s age (Figure 5). Overall, breastfeeding-related parameters had a greater relative influence on simulated POP levels from 6 months up to 45 months. For example, nearly half the variability in simulated blood POP levels at 6 months of age could be attributed to the influence of duration of breastfeeding, through first- or higher-order effects. As expected, weight gain during pregnancy had a greater influence on simulated levels at 6 months than at 16 or 45 months, whereas postpartum weight changes became more influential over time. The relative sensitivity of estimates to changes in the child’s weight was lower than the sensitivity to changes in breastfeeding and maternal weight parameters, except for birth weight, which had a relatively strong influence on simulated levels at 6 months. Prepregnancy body weight and gestational age at birth had little influence on estimates.

**Figure 6:**
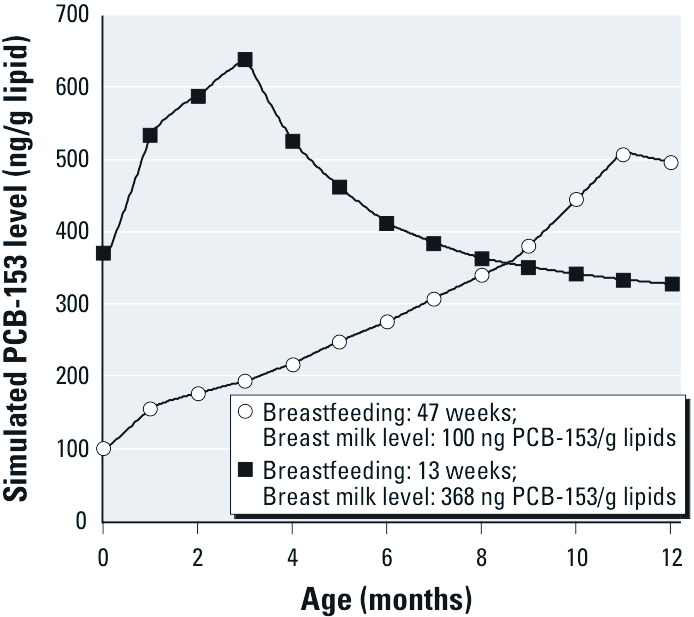
Blood PCB-153 profiles of two Inuit children for whom the commonly employed approach to estimate overall exposures suggests comparable exposure (specifically, weeks of exclusive breastfeeding × ng/g milk lipids = ~ 4,700).

*Exposure misclassification.* There was a striking difference between the simulated profiles of PCB-153 levels of two Inuit children with the same overall lactational exposure calculated with the commonly employed approach of multiplying milk levels by the duration of breastfeeding (Figure 6). This was especially true at 3 months of age, when blood PCB-153 level in the child exposed to the highest breast milk level (639 ng/g lipids) was more than three times that of the other child (193 ng/g lipids).

## Discussion

Adequate estimation of exposure to POPs through breastfeeding is critical for characterizing associations between postnatal exposure and children’s health in epidemiologic studies. Here, we developed a simple toxicokinetic model that can simulate complete children’s blood POP profiles based on cord blood, maternal blood, or breast milk levels and assessed its accuracy by comparing simulated blood levels to levels measured in children from two longitudinal birth cohorts. Using this model, we have shown that estimations based solely on breast milk levels and duration of breastfeeding may lead to exposure misclassification (Figure 6).

Model predictiveness ranged from weak (*R*^2^ = 0.10) to high (*R*^2^ = 0.83) depending on the cohort, the compound, the sample used to simulate children’s blood levels, and the age of the child when blood was collected for measurement. *R*^2^ values at 6 months of age were higher in the Inuit cohort than in the Slovak cohort, but *R*^2^ values cannot be compared across these studies because they may be influenced by sample size. Predictiveness also varied depending on the modeled chemical, although the compounds for which the model showed poorer accuracy differed between the Inuit (*p,p´-*DDT) and Slovak (*p,p´-*DDE and HCB) studies. Possible causes for divergence in model accuracy include population-specific physiologic characteristics, variability in the percentage of food intake attributable to breast milk during partial breastfeeding, and differences in analytic precision between laboratories.

*R*^2^ values also varied depending on the sample used to simulate children’s exposure. Simulations based on maternal blood concentrations at delivery generally gave the best predictions, closely followed by those based on cord blood. When simulations were based on breast milk concentrations collected approximately 1 month postpartum, *R*^2^ values were slightly lower and children’s blood levels were overestimated, likely because of methods used to adjust for lipids. Whereas lipids in breast milk are almost exclusively neutral triacyglycerol (Jensen 1999) in which POPs partition, blood contains phospholipids that bind lipophilic compounds with lower affinity (Poulin and Krishnan 1995). The usual method to adjust for blood lipids (Phillips et al. 1989) includes phospholipids and leads to a 40% overestimation of blood neutral lipids when compared with breast milk. This compares well with our 60% overestimation of children’s blood levels.

The model’s ability to estimate blood levels of children in the Slovak cohort decreased only slightly between 6 months and 45 months of age for most compounds, potentially because breastfeeding remains the main determinant of children’s blood levels throughout this period. In the same cohort of Inuits, Ayotte et al. (2003) could explain close to 50% of the variability in PCB-153 blood concentration in a group of 87 children at 6 months based solely on the duration of exclusive breastfeeding. When both maternal blood concentration and duration of breastfeeding were included in the regression model, this percentage increased to 66%. Similarly, Jacobson et al. (1989) could explain 60% of the variance in blood levels of 107 children at 4 years of age based on maternal milk PCB and weeks of breastfeeding, whereas children’s fish consumption had little relation to measured blood PCB levels. In 7-year-old Faroese children, the duration of breastfeeding was the primary predictor of blood POP levels, followed by body mass index and intake through diet (Barr et al. 2006). These studies concur with our results suggesting that, even at high dietary exposures, breastfeeding is a primary predictor of children’s blood POP levels up to 45 months of age.

Because epidemiologic questionnaires are invariably burdened with missing values and desired information is not systematically collected (e.g., weight loss postpartum), use of this toxicokinetic tool requires dealing with missing model inputs. In global sensitivity analyses, we showed that breastfeeding parameters are highly influential, so availability of information on breastfeeding is paramount to achieve precise estimations with the PBPK model. The absence of nursing information should therefore be considered an exclusion criterion in studies on postnatal exposure to POPs. Weight gain during pregnancy and weight changes after delivery also proved to be influential. Variations in body weight, which indicate fluctuations in maternal body lipids, have been shown to modulate POP levels in blood lipids and subcutaneous adipose tissue (Chevrier et al. 2000), thereby affecting breast milk levels and, in turn, children’s exposure. Using average weight variation during and after pregnancy, as we did in this validation study, likely reduces model accuracy. On the other hand, child weight, gestational age at birth, and prepregnancy maternal weight displayed a relatively small influence on children’s blood levels. Therefore, imputation of missing values can be performed without compromising model precision.

The model presented herein generates individualized exposure profiles from birth to 45 months of age from which blood POP levels can be determined for any period of interest [Figure 6; see also Supplemental Material, Figure S1 (http://dx.doi.org/10.1289/ehp.1205552)]. This is particularly useful when estimating the effects of POPs on systems that undergo specific developmental processes during constrained postnatal time frames, such as during brain development, with cell proliferation, differentiation, migration, synaptogenesis, apoptosis, and myelination occurring in different regions during precise periods (Rice and Barone 2000). Our recent study in Inuits supported this contention, because prenatal and postnatal exposures to PCBs were associated with different behavioral end points (Verner et al. 2010).

## Conclusions

The ability to detect associations between postnatal exposure to POPs and decrements in children’s health or development is tightly bound to our capacity to adequately estimate exposure during windows of susceptibility that are specific to the outcome of interest. Where such associations exist, the use of toxicokinetic modeling may facilitate their detection and characterization in epidemiologic studies.

## Supplemental Material

(197 KB) PDF

## References

[r1] Arcus-Arth A, Krowech G, Zeise L. (2005). Breast milk and lipid intake distributions for assessing cumulative exposure and risk.. J Expo Anal Environ Epidemiol.

[r2] Ayotte P, Muckle G, Jacobson JL, Jacobson SW, Dewailly É (2003). Assessment of pre- and postnatal exposure to polychlorinated biphenyls: lessons from the Inuit Cohort Study.. Environ Health Perspect.

[r3] Baker GL (1969). Human adipose tissue composition and age.. Am J Clin Nutr.

[r4] Barr DB, Weihe P, Davis MD, Needham LL, Grandjean P (2006). Serum polychlorinated biphenyl and organochlorine insecticide concentrations in a Faroese birth cohort.. Chemosphere.

[r5] Bitman J, Wood L, Hamosh M, Hamosh P, Mehta NR (1983). Comparison of the lipid composition of breast milk from mothers of term and preterm infants.. Am J Clin Nutr.

[r6] Boucher O, Burden MJ, Muckle G, Saint-Amour D, Ayotte P, Dewailly É (2012). Response inhibition and error monitoring during a visual go/no-go task in Inuit children exposed to lead, polychlorinated biphenyls, and methylmercury.. Environ Health Perspect.

[r7] Butte NF, Ellis KJ, Wong WW, Hopkinson JM, Smith EO (2003). Composition of gestational weight gain impacts maternal fat retention and infant birth weight.. Am J Obstet Gynecol.

[r8] Chevrier J, Dewailly É, Ayotte P, Mauriege P, Despres JP, Tremblay A (2000). Body weight loss increases plasma and adipose tissue concentrations of potentially toxic pollutants in obese individuals.. Int J Obes Relat Metab Disord.

[r9] Emond C, Charbonneau M, Krishnan K. (2005). Physiologically based modeling of the accumulation in plasma and tissue lipids of a mixture of PCB congeners in female Sprague-Dawley rats.. J Toxicol Environ Health A.

[r10] Fomon SJ, Haschke F, Ziegler EE, Nelson SE (1982). Body composition of reference children from birth to age 10 years.. Am J Clin Nutr.

[r11] Grandjean P, Budtz-Jorgensen E, Steuerwald U, Heinzow B, Needham LL, Jorgensen PJ (2003). Attenuated growth of breast-fed children exposed to increased concentrations of methylmercury and polychlorinated biphenyls.. FASEB J.

[r12] Haddad S, Poulin P, Krishnan K. (2000). Relative lipid content as the sole mechanistic determinant of the adipose tissue:blood partition coefficients of highly lipophilic organic chemicals.. Chemosphere.

[r13] Hertz-Picciotto I, Pastore LM, Beaumont JJ (1996). Timing and patterns of exposures during pregnancy and their implications for study methods.. Am J Epidemiol.

[r14] Hertz-Picciotto I, Trnovec T, Kocan A, Charles MJ, Ciznar P, Langer P (2003). PCBs and early childhood development in Slovakia: study design and background.. Fresenius Environ Bull.

[r15] Huisman M, Koopman-Esseboom C, Lanting CI, van der Paauw CG, Tuinstra LG, Fidler V (1995). Neurological condition in 18-month-old children perinatally exposed to polychlorinated biphenyls and dioxins.. Early Hum Dev.

[r16] ICRP (International Commission on Radiological Protection) (2002). Basic Anatomical and Physiological Data for Use in Radiological Protection: Reference Values. Publication 89.

[r17] Jacobson JL, Humphrey HE, Jacobson SW, Schantz SL, Mullin MD, Welch R (1989). Determinants of polychlorinated biphenyls (PCBs), polybrominated biphenyls (PBBs), and dichlorodiphenyl trichloroethane (DDT) levels in the sera of young children.. Am J Public Health.

[r18] Jacobson JL, Jacobson SW (1996). Intellectual impairment in children exposed to polychlorinated biphenyls *in utero*.. N Engl J Med.

[r19] Jensen RG (1999). Lipids in human milk.. Lipids.

[r20] Jorissen J. (2007). Literature review. Outcomes associated with postnatal exposure to polychlorinated biphenyls (PCBs) via breast milk.. Adv Neonatal Care.

[r21] Kent JC, Mitoulas L, Cox DB, Owens RA, Hartmann PE (1999). Breast volume and milk production during extended lactation in women.. Exp Physiol.

[r22] Koopman-Esseboom C, Weisglas-Kuperus N, de Ridder MA, Van der Paauw CG, Tuinstra LG, Sauer PJ (1996). Effects of polychlorinated biphenyl/dioxin exposure and feeding type on infants’ mental and psychomotor development.. Pediatrics.

[r23] Kramer MS Platt RW Wen SW Joseph KS Allen A Abrahamowicz M 2001 A new and improved population-based Canadian reference for birth weight for gestational age. Pediatrics 108 2 e35; doi: [Online 15 October 2001] 10.1542/peds.108.2.e35 11483845

[r24] Kuczmarski RJ, Ogden CL, Grummer-Strawn LM, Flegal KM, Guo SS, Wei R, et al (2000). CDC Growth Charts: United States. Advance Data from Vital and Health Statistics.

[r25] Lanting CI, Fidler V, Huisman M, Boersma ER (1998). Determinants of polychlorinated biphenyl levels in plasma from 42-month-old children.. Arch Environ Contam Toxicol.

[r26] McDowell MA, Fryar CD, Hirsh R, Ogden CL (2005). Anthropometric Reference Data for Children and Adults: U.S. Population, 1999–2002. Advance Data from Vital and Health Statistics.

[r27] McLachlan MS (1993). Digestive tract absorption of polychlorinated dibenzo-*p*-dioxins, dibenzofurans, and biphenyls in a nursing infant.. Toxicol Appl Pharmacol.

[r28] McNally K Cotton R Loizou GD 2011 A workflow for global sensitivity analysis of PBPK models. Front Pharmacol 2 31; doi:10.3389/fphar.2011.00031 [Online 23 June 2011] 21772819 PMC3128931

[r29] Muckle G, Ayotte P, Dewailly É, Jacobson SW, Jacobson JL (2001). Prenatal exposure of the northern Quebec Inuit infants to environmental contaminants.. Environ Health Perspect.

[r30] Pan IJ, Daniels JL, Goldman BD, Herring AH, Siega-Riz AM, Rogan WJ (2009). Lactational exposure to polychlorinated biphenyls, dichlorodiphenyltrichloroethane, and dichlorodiphenyldichloroethylene and infant neurodevelopment: an analysis of the pregnancy, infection, and nutrition babies study.. Environ Health Perspect.

[r31] Patandin S, Weisglas-Kuperus N, de Ridder MA, Koopman-Esseboom C, van Staveren WA, van der Paauw CG (1997). Plasma polychlorinated biphenyl levels in Dutch preschool children either breast-fed or formula-fed during infancy.. Am J Public Health.

[r32] Phillips DL, Pirkle JL, Burse VW, Bernert JT, Henderson LO, Needham LL (1989). Chlorinated hydrocarbon levels in human serum: effects of fasting and feeding.. Arch Environ Contam Toxicol.

[r33] Plusquellec P, Muckle G, Dewailly É, Ayotte P, Begin G, Desrosiers C (2010). The relation of environmental contaminants exposure to behavioral indicators in Inuit preschoolers in Arctic Quebec.. Neurotoxicology.

[r34] Poulin P, Krishnan K. (1995). An algorithm for predicting tissue: blood partition coefficients of organic chemicals from n-octanol: water partition coefficient data.. J Toxicol Environ Health.

[r35] Ribas-Fito N, Grimalt JO, Marco E, Sala M, Mazon C, Sunyer J (2005). Breastfeeding and concentrations of HCB and *p,p*´-DDE at the age of 1 year.. Environ Res.

[r36] Rice D, Barone S (2000). Critical periods of vulnerability for the developing nervous system: evidence from humans and animal models.. Environ Health Perspect.

[r37] Ritter R, Scheringer M, MacLeod M, Moeckel C, Jones KC, Hungerbuhler K (2011). Intrinsic human elimination half-lives of polychlorinated biphenyls derived from the temporal evolution of cross-sectional biomonitoring data from the United Kingdom.. Environ Health Perspect.

[r38] Rogan WJ, Gladen BC, McKinney JD, Carreras N, Hardy P, Thullen J (1987). Polychlorinated biphenyls (PCBs) and dichlorodiphenyl dichloroethene (DDE) in human milk: effects on growth, morbidity, and duration of lactation.. Am J Public Health.

[r39] Rossner S. (1997). Weight gain in pregnancy.. Hum Reprod.

[r40] Saint-Amour D, Roy MS, Bastien C, Ayotte P, Dewailly É, Despres C (2006). Alterations of visual evoked potentials in preschool Inuit children exposed to methylmercury and polychlorinated biphenyls from a marine diet.. Neurotoxicology.

[r41] Salmenpera L, Perheentupa J, Siimes MA (1985). Exclusively breast-fed healthy infants grow slower than reference infants.. Pediatr Res.

[r42] Smith D. (1999). Worldwide trends in DDT levels in human breast milk.. Int J Epidemiol.

[r43] Sohlstrom A, Forsum E. (1995). Changes in adipose tissue volume and distribution during reproduction in Swedish women as assessed by magnetic resonance imaging.. Am J Clin Nutr.

[r44] To-Figueras J, Barrot C, Sala M, Otero R, Silva M, Ozalla MD (2000). Excretion of hexachlorobenzene and metabolites in feces in a highly exposed human population.. Environ Health Perspect.

[r45] Verner MA, Plusquellec P, Muckle G, Ayotte P, Dewailly É, Jacobson SW (2010). Alteration of infant attention and activity by polychlorinated biphenyls: unravelling critical windows of susceptibility using physiologically based pharmacokinetic modeling.. Neurotoxicology.

[r46] Wolff MS, Zeleniuch-Jacquotte A, Dubin N, Toniolo P (2000). Risk of breast cancer and organochlorine exposure.. Cancer Epidemiol Biomarkers Prev.

