# Supplementary material for: Toxicokinetic Modeling of Persistent Organic Pollutant Levels in Blood from Birth to 45 Months of Age in Longitudinal Birth Cohort Studies
Source: Environ Health Perspect. 2012 Oct 17;121(1):131–7. doi: 10.1289/ehp.1205552 (PMC3553439; doi:10.1289/ehp.1205552)
Supplement: (197 KB) PDF [file ehp.1205552.s001.pdf]

SUPPLEMENTAL MATERIAL

Title: Toxicokinetic Modeling of Persistent Organic Pollutant Levels in Blood from Birth to 45 Months of Age in Longitudinal Birth Cohort Studies

Authors: Marc-André Verner, Dean Sonneborn, Kinga Lancz, Gina Muckle, Pierre Ayotte, Éric Dewailly, Anton Kocan, Ľubica Palkovičová, Tomas Trnovec, Sami Haddad, Irva Hertz-Picciotto, Merete Eggesbø

Table of contents

Figure S1. Examples of simulated toxicokinetic profiles in children breastfed for 0, 4 and 12 months. Simulations were carried out using a daily dose of 5 ng PCB-153/kg/d in mothers 2
Instructions for the toxicokinetic model 3
Toxicokinetic model code (csl and m files)..6

Figure S1. Examples of simulated toxicokinetic profiles in children breastfed for 0, 4 and 12 months. Simulations were carried out using a daily dose of 5 ng PCB-153/kg/d in mothers.

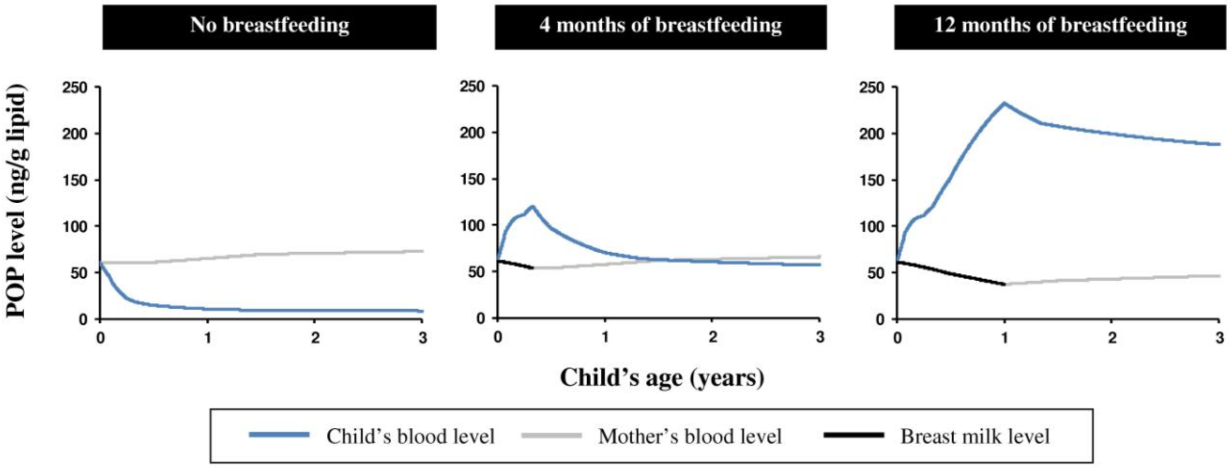

The model predicted that POP levels in children who were not breastfed dropped drastically after birth as the initial body burden acquired *in utero* was diluted due to growth. Breastfeeding led to sharp increases in simulated children's POP levels, followed by a decrease at the time of weaning. The decrease was more accentuated when nursing was stopped after 4 months than after 12 months, which is consistent with decreasing growth rates after 12 months of age. Because POPs are extensively excreted through breast milk, simulated maternal POP levels declined during the period of breastfeeding despite the postpartum weight loss which has the opposite effect due to a decrease in the volume of distribution.

Instructions for the toxicokinetic model

1. Open acslX.
2. Create a new workspace from the start page.
3. In the Project Files section, right-click on Model Files > Add new > CSL file. Copy-paste the content of the **CSL FILE** section of the Supplemental Material.
4. In the Project Files section, right-click on Runtime Files > Add new > M file. Copy-paste the content of the **M FILE** section of the Supplemental Material.

To run the model for individuals in a dataset

1. Create your dataset in Excel (save in the .xls format [Excel 97-2003 Workbook in the "Save as" options]). The name must not contain spaces (e.g., Dataset.xls). Use the framework defined in the .m file to configure the columns in your Excel spreadsheet. There must be a value in each of the cells. Should there be missing values, you must either impute the value or exclude the individual.
   a) *Individuals:* This variable identifies the individuals in your dataset. The first individual has a value of 1, the second has a value of 2, and so on.
   b) *ID:* Study ID. This variable allows you to merge the simulated results to your original dataset.
   c) *Age_delivery:* Mother's age at delivery. It needs to be in years with at least 3 decimals (e.g., 25.764).
   d) *Prepregnancy_bw:* Mother's pre-pregnancy body weight in kg.
   e) *Weight_gain_pregnancy:* Weight gain at the end of pregnancy (kg). If this variable was not collected, you could use an average value of 14.5 kg (Butte et al. 2003).
   f) *Postpartum_weight_1 and _2:* Mother's weight at first (1) and second (2) postpartum measurement (kg). If these variables were not collected, you could assume that body weight was 2 kg above pre-pregnancy weight 0.5 year after delivery (Butte et al. 2003) and back to pre-pregnancy weight by the end of the first year (arbitrary).
   g) *Age_postpartum_weight_1 and _2:* Mother's age at postpartum weight measurements. Refer to "f) *Postpartum_weight*" description if this information was not collected.

h) *Sex:* Child sex (0: female, 1: male)

i) *Gestational_age:* Gestational age (year). Should this information be unavailable, you could use an average value of 0.75 year (9 months).

j) *Birthweight:* Birth weight (kg). If this information is missing you could use 3.5 kg for males or 3.4 kg for females (Kuczmarski et al. 2000).

k) *Child_weight_1*, *_2* and *_3:* Child's weight at first (1), second (2) and third (3) measurements. If this information is missing, you could use 7.8, 12.7 and 18.0 kg for males or 7.2, 12.1, 18.0 kg for females at 0.5, 2.0 and 5.0 years of age (Kuczmarski et al. 2000).

l) *Age_child_weight_1*, *_2* and *_3:* Age of child at weight measurements. Refer to "k) *Child_weight*" description if this information was not collected.

m) *Duration_excl_breastfeeding:* Duration of exclusive breastfeeding (years).

n) *Duration_total_breastfeeding:* Duration of total breastfeeding including exclusive and partial breast-feeding (years).

o) *Fraction_bf:* Fraction of food intake attributable to breast milk during partial breastfeeding. A value of 0.5 could be used when this information is not available.

p) *POP level:* Level of persistent organic pollutant in either maternal blood, cord blood or breast milk lipids (ug/kg lipids or ng/g lipids).

q) *Mother age at sample:* Age of mother when maternal blood, cord blood or breast milk sample was drawn. It needs to be in years with at least 3 decimals (e.g., 25.764).

r) *Compound:* Chemical to be modeled (1: PCB52, 2: PCB105, 3: PCB118, 4: PCB138, 5: PCB153, 6: PCB170, 7: PCB180, 8: PCB187, 9: DDE, 10: DDT, 11: HCB).

2. In the xlsread commands in the .m file, specify the path to the Excel file, the sheet you want to read data from and the arrays for each variable. For example:
   Individual = xlsread("C:\Dataset.xls", "Sheet1", "A2:A500")
3. Create an Excel spreadsheet to save results (save in the .xls format [Excel 97-2003 Workbook in the "Save as" options]) (e.g., Results.xls).
4. Specify the number of individuals in the dataset (variable "NumIndividuals" in the .m file)

5. In the xlswrite command at the end of the .m file, specify the path to the Excel file, the sheet you want to write data in and the array in which results will be written. For example:
   xlswrite("C:\Results.xls", "Sheet1", "A2:A500", calculated_c)
   Data will be placed in the columns according to the order of variables in the *calculated_c* array in the .m file.
6. Save the changes by right-clicking on the Verner_2012_ script.m file under the "Runtime Files" heading and selecting "Save".
7. Build the .csl model (Ctrl+Shift+B).
8. Start the run by right-clicking on the Verner_2012_ script.m file under the "Runtime Files" heading and selecting "Run".
9. Information on the number of runs completed can be seen in the "Command Window". Make sure the Excel file in which data will be written is closed by the end of the simulations (acslX cannot write data if the Excel file is open). The results are only available after all the simulations are done.

References

Butte NF, Ellis KJ, Wong WW, Hopkinson JM, Smith EO. 2003. Composition of gestational weight gain impacts maternal fat retention and infant birth weight. Am J Obstet Gynecol 189(5): 1423-1432.

Kuczmarski RJ, Ogden CL, Grummer-Strawn LM, Flegal KM, Guo SS, Wei R, et al. 2000. CDC growth charts: United States. Advance data from vital and health statistics. Hyattsville, MD.

CSL FILE

```
PROGRAM

!***********************************!
!***********************************!
!**   POP toxicokinetic model     **!
!**   Coded by Marc-Andre Verner  **!
!**                               **!
!**   Units                       **!
!**   Time: hours                 **!
!**   Volumes: L                  **!
!**   Quantities: ug              **!
!***********************************!
!***********************************!


INITIAL    ! Start of initial section

!*****************************************************************************
! Individual information
! These can be imported for individuals from
! Excel spreadsheets using the script
! Verner_2012_script.m

! Dyad ID
CONSTANT ID                          = 2005   ! ID number

! Maternal variables
CONSTANT AGE_delivery                = 25.634 ! Age of mother at delivery (years)
CONSTANT PREPREGNANCY_BW             = 61.0   ! Pre-pregnancy body weight (kg)
CONSTANT WEIGHT_GAIN_PREGNANCY       = 12.5   ! Weight gain during pregnancy (kg)
CONSTANT POSTPARTUM_WEIGHT_1         = 63.0   ! Postpartum weight at first weighing (kg)
CONSTANT POSTPARTUM_WEIGHT_2         = 62.0   ! Postpartum weight at second weighing (kg)
CONSTANT AGE_POSTPARTUM_WEIGHT_1     = 26.134 ! Age of mother at first postpartum weighing (years)
CONSTANT AGE_POSTPARTUM_WEIGHT_2     = 26.634 ! Age of mother at second postpartum weighing (years)

! Child variables
CONSTANT SEX                         = 1      ! Child sex (0: female, 1: male)
CONSTANT GESTATIONAL_AGE             = 0.76   ! Gestational age at birth (years)
CONSTANT BIRTHWEIGHT                 = 3.3    ! Child birth weight (kg)
CONSTANT CHILD_WEIGHT_1              = 7.75   ! Child weight at 1st measurement
CONSTANT CHILD_WEIGHT_2              = 12.6   ! Child weight at 2nd measurement
CONSTANT CHILD_WEIGHT_3              = 18.2   ! Child weight at 3rd measurement
CONSTANT AGE_CHILD_WEIGHT_1          = 0.521  ! Child age at first weight measurement (years)
CONSTANT AGE_CHILD_WEIGHT_2          = 1.333  ! Child age at second weight measurement (years)
CONSTANT AGE_CHILD_WEIGHT_3          = 4.246  ! Child age at third weight measurement (years)
```

```
! Breast-feeding variables
CONSTANT DURATION_EXCL_Breastfeeding  = 0.5    ! Duration of exclusive breastfeeding (years)
CONSTANT DURATION_TOTAL_Breastfeeding = 0.5    ! Total duration breastfeeding (years)
CONSTANT FRACTION_BF_1m               = 0.5    ! Fraction of food intake attributable to breast milk (month 1)
CONSTANT FRACTION_BF_2m               = 0.5    ! Fraction of food intake attributable to breast milk (month 2)
CONSTANT FRACTION_BF_3m               = 0.5    ! Fraction of food intake attributable to breast milk (month 3)
CONSTANT FRACTION_BF_4m               = 0.5    ! Fraction of food intake attributable to breast milk (month 4)
CONSTANT FRACTION_BF_5m               = 0.5    ! Fraction of food intake attributable to breast milk (month 5)
CONSTANT FRACTION_BF_6m               = 0.5    ! Fraction of food intake attributable to breast milk (month 6)
CONSTANT FRACTION_BF_7m               = 0.5    ! Fraction of food intake attributable to breast milk (month 7)
CONSTANT FRACTION_BF_8m               = 0.5    ! Fraction of food intake attributable to breast milk (month 8)
CONSTANT FRACTION_BF_9m               = 0.5    ! Fraction of food intake attributable to breast milk (month 9)
CONSTANT FRACTION_BF_10m              = 0.5    ! Fraction of food intake attributable to breast milk (month 10)
CONSTANT FRACTION_BF_11m              = 0.5    ! Fraction of food intake attributable to breast milk (month 11)
CONSTANT FRACTION_BF_12m              = 0.5    ! Fraction of food intake attributable to breast milk (month 12)
CONSTANT FRACTION_BF_13m              = 0.5    ! Fraction of food intake attributable to breast milk (month 13)
CONSTANT FRACTION_BF_14m              = 0.5    ! Fraction of food intake attributable to breast milk (month 14)
CONSTANT FRACTION_BF_15m              = 0.5    ! Fraction of food intake attributable to breast milk (month 15)
CONSTANT FRACTION_BF_16m              = 0.5    ! Fraction of food intake attributable to breast milk (month 16)
CONSTANT FRACTION_BF_17m              = 0.5    ! Fraction of food intake attributable to breast milk (month 17)
CONSTANT FRACTION_BF_18m              = 0.5    ! Fraction of food intake attributable to breast milk (month 18)
CONSTANT FRACTION_BF_19m              = 0.5    ! Fraction of food intake attributable to breast milk (month 19)
CONSTANT FRACTION_BF_20m              = 0.5    ! Fraction of food intake attributable to breast milk (month 20)
CONSTANT FRACTION_BF_21m              = 0.5    ! Fraction of food intake attributable to breast milk (month 21)
CONSTANT FRACTION_BF_22m              = 0.5    ! Fraction of food intake attributable to breast milk (month 22)
CONSTANT FRACTION_BF_23m              = 0.5    ! Fraction of food intake attributable to breast milk (month 23)
CONSTANT FRACTION_BF_24m              = 0.5    ! Fraction of food intake attributable to breast milk (month 24)

! Dosing variable
CONSTANT DOSE                         = 0.01   ! Daily oral dose (ug/kg)
CONSTANT COMPOUND                     = 5      ! 1:PCB52, 2:PCB105, 3:PCB118, 4:PCB138, 5:PCB153,
                                               ! 6:PCB170, 7:PCB180, 8:PCB187, 9:DDE, 10:DDT, 11:HCB
! Sample variables
CONSTANT MOTHER_AGE_SAMPLE            = 23     ! Age of mother at blood/cord/milk sample (years)
CONSTANT POP_LEVEL                    = 116    ! Mother fat pop level (ug/kg lipid)

END ! End of initial section


DYNAMIC ! Start of dynamic sections


!**********************************************************************************
! Length of simulations

TSTOPage = AGE_delivery + 5       ! Length of simulation / mother's age (yrs)
```

```
TSTOP = 24.*365.*TSTOPage         ! Length of simulation (hrs)
POINTS = 300                      ! Number of points in plot
schedule stopthesim .at. TSTOP    ! Schedule end of simulation
discrete stopthesim               ! When simulation reaches stopthesim event...
termt(.true.)                     ! ...terminate simulation
end                               ! End of discrete event



!*********************************************************************************
! Simulation parameters

ALGORITHM IALG = 2         ! Use Gear algorithm
MAXTERVAL MAXT = 24        ! Maximum interval for integrations
MINTERVAL MINT = 1.0       ! Minimal interval for integrations
CINTERVAL CINT = 24        ! Communication interval


DERIVATIVE    ! Start of DERIVATIVE SECTION

!*********************************************************************************
! Age at conception

AGE_CONCEPTION = AGE_DELIVERY-GESTATIONAL_AGE          ! Mother's age at conception (years)


!*********************************************************************************
! Basal body weight
! (not taking pregnancy weight changes into account)
! Curves are based on 50th percentile charts and adjusted for individual measurements

! Mother
table table_bw_mother,1,10/0.0, 0.5, 1.0, 2.0, 5.0, 10.0, 13.0, 16.0, 20.0, 100.0, 3.4, 7.2, 9.5, 12.1, &
                      18.0, 33.0, 46.0, 54.0, 58.0, 58.0/
Diff_profile = (1-Switch_conception)*(AGE*(PREPREGNANCY_BW-table_bw_mother(Age_conception))/Age_conception) &
               +Switch_conception*(PREPREGNANCY_BW-table_bw_mother(Age_conception))
BW = Diff_profile + table_bw_mother(Age)

! Child
table table_bw_males  ,1,10/0.0, 0.5, 1.0, 2.0, 5.0, 10.0, 13.0, 16.0, 20.0, 100.0, 3.5, 7.8, 10.3, 12.7, &
                       18.0, 32.0, 46.0, 61.0, 71.0, 71.0/
table table_bw_females,1,10/0.0, 0.5, 1.0, 2.0, 5.0, 10.0, 13.0, 16.0, 20.0, 100.0, 3.4, 7.2, 9.5, 12.1, &
                       18.0, 33.0, 46.0, 54.0, 58.0, 58.0/
NORM_BW_CHILD     = sex*table_bw_males(Age_child)+(1-sex)*table_bw_females(Age_child)
Diff_norm_birth   = BIRTHWEIGHT-(sex*3.5 + (1-sex)*3.4)
Diff_norm_WEIGHT1 = CHILD_WEIGHT_1-(sex*table_bw_males(AGE_CHILD_WEIGHT_1)+(1-sex)*table_bw_females(AGE_CHILD_WEIGHT_1))
Diff_norm_WEIGHT2 = CHILD_WEIGHT_2-(sex*table_bw_males(AGE_CHILD_WEIGHT_2)+(1-sex)*table_bw_females(AGE_CHILD_WEIGHT_2))
```

```
Diff_norm_WEIGHT3 = CHILD_WEIGHT_3-(sex*table_bw_males(AGE_CHILD_WEIGHT_3)+(1-sex)*table_bw_females(AGE_CHILD_WEIGHT_3))
Diff_profile_child = (SWITCH_DELIVERY-SWITCH_AGE_CHILD_WEIGHT1)*(Diff_norm_birth+AGE_child*(Diff_norm_WEIGHT1-
Diff_norm_birth)/AGE_CHILD_WEIGHT_1) &
                    +(SWITCH_AGE_CHILD_WEIGHT1-SWITCH_AGE_CHILD_WEIGHT2)*(Diff_norm_WEIGHT1+(Age_child-AGE_CHILD_WEIGHT_1) &
                    *(Diff_norm_WEIGHT2-Diff_norm_WEIGHT1)/(AGE_CHILD_WEIGHT_2-AGE_CHILD_WEIGHT_1)) &
                    +(SWITCH_AGE_CHILD_WEIGHT2-SWITCH_AGE_CHILD_WEIGHT3)*(Diff_norm_WEIGHT2+(Age_child-AGE_CHILD_WEIGHT_2) &
                    *(Diff_norm_WEIGHT3-Diff_norm_WEIGHT2)/(AGE_CHILD_WEIGHT_3-AGE_CHILD_WEIGHT_2)) &
                    +SWITCH_AGE_CHILD_WEIGHT3*Diff_norm_WEIGHT3
BW_child =Diff_profile_child+NORM_BW_CHILD




! Fetus
table table_fetal_weight,1,7/0.0, 0.217, 0.353, 0.463, 0.551, 0.639, 0.768, 0.0, 0.0, 0.26, 0.69, 1.25, 2.02, 3.28/
NORM_BIRTHWEIGHT_FOR_GA   = table_fetal_weight(GESTATIONAL_AGE)
RATIO_BIRTHWEIGHT_NORM    = BIRTHWEIGHT/NORM_BIRTHWEIGHT_FOR_GA
FETAL_WEIGHT              = (SWITCH_CONCEPTION-SWITCH_DELIVERY)*RATIO_BIRTHWEIGHT_NORM*table_fetal_weight(FETAL_AGE)


!*************************************************************************************
! BODY FAT
! Based on data from
! Fomon et al. 1982
! and ICRP 2002

! Mother
table bodyfat_mother,1,17/0.0,0.08,0.17,0.25,0.33,0.5,1,2,4,6,8,10,12,16,29,39,69,0.15,0.16,0.21, &
                     0.24,0.25,0.26,0.24, 0.20,0.17,0.16,0.17,0.19,0.23,0.26,0.29,0.31,0.41/
FAT_GAIN_PREGNANCY      = 0.75*(WEIGHT_GAIN_PREGNANCY - Birthweight-5.8)
SLOPE_GAIN_PREGNANCY    = (SWITCH_CONCEPTION-SWITCH_DELIVERY)*(FAT_GAIN_PREGNANCY*(AGE-AGE_CONCEPTION)/GESTATIONAL_AGE)
FAT_POSTPARTUM_WEIGHT_1 = POSTPARTUM_WEIGHT_1-PREPREGNANCY_BW
FAT_POSTPARTUM_WEIGHT_2 = POSTPARTUM_WEIGHT_2-PREPREGNANCY_BW
SLOPE_LOSS_POSTPARTUM   = (SWITCH_DELIVERY-SWITCH_POSTPARTUM_WEIGHT_1)&
                        *(FAT_GAIN_PREGNANCY-(FAT_GAIN_PREGNANCY-FAT_POSTPARTUM_WEIGHT_1) &
                        *(AGE-AGE_DELIVERY)/(AGE_POSTPARTUM_WEIGHT_1-AGE_DELIVERY)) &
                        +(SWITCH_POSTPARTUM_WEIGHT_1-SWITCH_POSTPARTUM_WEIGHT_2)&
                        *(FAT_POSTPARTUM_WEIGHT_1-(FAT_POSTPARTUM_WEIGHT_1-FAT_POSTPARTUM_WEIGHT_2) &
                        *(AGE-AGE_POSTPARTUM_WEIGHT_1)/(AGE_POSTPARTUM_WEIGHT_2-AGE_POSTPARTUM_WEIGHT_1))&
                        + SWITCH_POSTPARTUM_WEIGHT_2*FAT_POSTPARTUM_WEIGHT_2
VOL_FAT_MOTHER          = (BW*bodyfat_mother(age))+SLOPE_GAIN_PREGNANCY+SLOPE_LOSS_POSTPARTUM

table bodyfat_female,1,17/0.0,0.08,0.17,0.25,0.33,0.5,1,2,4,6,8,10,12,16,29,39,69,0.15,0.16,0.21, &
                     0.24,0.25,0.26, 0.24,0.20,0.17,0.16,0.17,0.19,0.23,0.26,0.29,0.31,0.41/
table bodyfat_male  ,1,17/0.0,0.08,0.17,0.25,0.33,0.5,1,2,4,6,8,10,12,16,21,36,69,0.14,0.15,0.19, &
                     0.23,0.25,0.25, 0.23,0.20,0.16,0.14,0.13,0.14,0.17,0.16,0.17,0.20,0.33/
```

```
VOL_FAT_CHILD  = (SWITCH_CONCEPTION-SWITCH_DELIVERY)*FETAL_WEIGHT*(sex*0.14+(1-sex)*0.15)&
                 +SWITCH_DELIVERY*(BW_child*(sex*bodyfat_male(age_child) + (1-sex)*bodyfat_female(age_child))) &
                 +0.001


!**********************************************************************************
! Breast-feeding
! Data from Salmenpera 1985
! and Kent 1999

! Volume of milk ingested (L/H)
VOLUME_MILK_excl_0_12m = (BW_child*(-0.0024*Age_child + 0.0063))                       ! Milk consumption during exclusive breast-
feeding (l/h)(Salmenpera et al. 1985)
VOLUME_MILK_12_24m     = ((-0.2049*AGE_child)+0.4508)/24                               ! Milk consumption from 12 up to 24 months
(l/h)(Kent 1999)
FRACTION_BF            = (SWITCH_DELIVERY-SWITCH_CHILD_1m)*FRACTION_BF_1m &            ! Fraction of food intake attributable to
breast-feeding
                         +(SWITCH_CHILD_1m-SWITCH_CHILD_2m)*FRACTION_BF_2m &
                         +(SWITCH_CHILD_2m-SWITCH_CHILD_3m)*FRACTION_BF_3m &
                         +(SWITCH_CHILD_3m-SWITCH_CHILD_4m)*FRACTION_BF_4m &
                         +(SWITCH_CHILD_4m-SWITCH_CHILD_5m)*FRACTION_BF_5m &
                         +(SWITCH_CHILD_5m-SWITCH_CHILD_6m)*FRACTION_BF_6m &
                         +(SWITCH_CHILD_6m-SWITCH_CHILD_7m)*FRACTION_BF_7m &
                         +(SWITCH_CHILD_7m-SWITCH_CHILD_8m)*FRACTION_BF_8m &
                         +(SWITCH_CHILD_8m-SWITCH_CHILD_9m)*FRACTION_BF_9m &
                         +(SWITCH_CHILD_9m-SWITCH_CHILD_10m)*FRACTION_BF_10m &
                         +(SWITCH_CHILD_10m-SWITCH_CHILD_11m)*FRACTION_BF_11m &
                         +(SWITCH_CHILD_11m-SWITCH_CHILD_12m)*FRACTION_BF_12m &
                         +(SWITCH_CHILD_12m-SWITCH_CHILD_13m)*FRACTION_BF_13m &
                         +(SWITCH_CHILD_13m-SWITCH_CHILD_14m)*FRACTION_BF_14m &
                         +(SWITCH_CHILD_14m-SWITCH_CHILD_15m)*FRACTION_BF_15m &
                         +(SWITCH_CHILD_15m-SWITCH_CHILD_16m)*FRACTION_BF_16m &
                         +(SWITCH_CHILD_16m-SWITCH_CHILD_17m)*FRACTION_BF_17m &
                         +(SWITCH_CHILD_17m-SWITCH_CHILD_18m)*FRACTION_BF_18m &
                         +(SWITCH_CHILD_18m-SWITCH_CHILD_19m)*FRACTION_BF_19m &
                         +(SWITCH_CHILD_19m-SWITCH_CHILD_20m)*FRACTION_BF_20m &
                         +(SWITCH_CHILD_20m-SWITCH_CHILD_21m)*FRACTION_BF_21m &
                         +(SWITCH_CHILD_21m-SWITCH_CHILD_22m)*FRACTION_BF_22m &
                         +(SWITCH_CHILD_22m-SWITCH_CHILD_23m)*FRACTION_BF_23m &
                         +(SWITCH_CHILD_23m-SWITCH_CHILD_24m)*FRACTION_BF_24m

VOLUME_MILK = (SWITCH_DELIVERY-SWITCH_END_EXCLUSIVE_BREASTFEEDING)* &                   ! Switch exclusive breast-feeding (I/O)
              ((1-SWITCH_CHILD_12m)*VOLUME_MILK_excl_0_12m &                           ! Volume exclusive bf 0-12 months (l/h)
              +(SWITCH_CHILD_12m-SWITCH_CHILD_24m)*VOLUME_MILK_12_24m ) &              ! Volume 12-24 months (l/h)
              +(SWITCH_END_EXCLUSIVE_BREASTFEEDING-SWITCH_END_TOTAL_BREASTFEEDING)*&   ! Switch partial breast-feeding (I/O)
              FRACTION_BF*(((1-SWITCH_CHILD_12m)*VOLUME_MILK_excl_0_12m &              ! Fraction exclusive bf 0-12 months (l/h)
              +(SWITCH_CHILD_12m-SWITCH_CHILD_24m)*VOLUME_MILK_12_24m ))               ! Fraction bf 12-24 months (l/h)
```

```
! Fraction of lipids in breast milk (taken from Verner et al. 2009)
FRACTION_LIPIDS_MILK = (SWITCH_DELIVERY-SWITCH_END_TOTAL_BREASTFEEDING)*&
                       (0.0034*LOG(max (0.00001, AGE-Age_delivery))+0.0414)


!*************************************************************************************
! Elimination

initial
CONSTANT HALF_LIFE_PCB52   = 22776  ! PCB-52 Half-life (hours)  ** Ritter et al. 2011
CONSTANT HALF_LIFE_PCB105  = 45552  ! PCB-105 Half-life (hours) ** Ritter et al. 2011
CONSTANT HALF_LIFE_PCB118  = 81468  ! PCB-118 Half-life (hours) ** Ritter et al. 2011
CONSTANT HALF_LIFE_PCB138  = 94608  ! PCB-138 Half-life (hours) ** Ritter et al. 2011
CONSTANT HALF_LIFE_PCB153  = 126144 ! PCB-153 Half-life (hours) ** Ritter et al. 2011
CONSTANT HALF_LIFE_PCB170  = 135780 ! PCB-170 Half-life (hours) ** Ritter et al. 2011
CONSTANT HALF_LIFE_PCB180  = 100740 ! PCB-180 Half-life (hours) ** Ritter et al. 2011
CONSTANT HALF_LIFE_PCB187  = 91980  ! PCB-187 Half-life (hours) ** Ritter et al. 2011
CONSTANT HALF_LIFE_DDE     = 113880 ! DDE Half-life (hours)     ** Wolfe et al. 2000
CONSTANT HALF_LIFE_DDT     = 43800  ! DDT Half-life (hours)     ** Smith et al. 1999
CONSTANT HALF_LIFE_HCB     = 52560  ! HCB Half-life (hours)     ** To-Figueras et al. 1997
end


if(compound .eq. 1) then
HALF_LIFE = HALF_LIFE_PCB52
else if(compound .eq. 2) then
HALF_LIFE = HALF_LIFE_PCB105
else if(compound .eq. 3) then
HALF_LIFE = HALF_LIFE_PCB118
else if(compound .eq. 4) then
HALF_LIFE = HALF_LIFE_PCB138
else if(compound .eq. 5) then
HALF_LIFE = HALF_LIFE_PCB153
else if(compound .eq. 6) then
HALF_LIFE = HALF_LIFE_PCB170
else if(compound .eq. 7) then
HALF_LIFE = HALF_LIFE_PCB180
else if(compound .eq. 8) then
HALF_LIFE = HALF_LIFE_PCB187
else if(compound .eq. 9) then
HALF_LIFE = HALF_LIFE_DDE
else if(compound .eq. 10) then
HALF_LIFE = HALF_LIFE_DDT
else if(compound .eq. 11) then
HALF_LIFE = HALF_LIFE_HCB
end if
```

```
!*********************************************************************************
! Rates of transfer

RATE_MILK_LIPIDS_INTAKE = SWITCH_BREASTFEEDING*VOLUME_MILK*FRACTION_LIPIDS_MILK     ! Rate of milk lipid intake (l lipids/h)
BREASTMILK_TRANSFER     = C_MOTHER*RATE_MILK_LIPIDS_INTAKE                          ! Rate of POP intake in children (ug/h)
RAO                     = BW*DOSE/24                                                ! Rate of POP intake in mothers (ug/h)
ELIMINATION             = A_MOTHER*log(2)/HALF_LIFE                                 ! Elimination rate in mothers
ELIMINATION_child       = SWITCH_DELIVERY*(A_CHILD*log(2)/HALF_LIFE)                ! Elimination rate in children
MOTHER_FETUS_TRANSFER   = (SWITCH_CONCEPTION-SWITCH_DELIVERY)*(C_MOTHER)            ! Placental diffusion from mother to fetus
FETUS_MOTHER_TRANSFER   = (SWITCH_CONCEPTION-SWITCH_DELIVERY)*(C_CHILD)             ! Placental diffusion from fetus to mother


!*********************************************************************************
! Age

RAGE = 1/24./365.                                          ! Rate of increase in mother's age (years/h)
AGE = INTEG(RAGE, 0.)                                      ! Age of mother (years)
AGE_child = SWITCH_DELIVERY*(AGE-AGE_delivery)             ! Age of child (years)
FETAL_AGE = SWITCH_CONCEPTION*(AGE-AGE_CONCEPTION)         ! Age of fetus (years)


!*********************************************************************************
! SWTICHES

initial
SWITCH_CHILD_1m                  = 0
SWITCH_CHILD_2m                  = 0
SWITCH_CHILD_3m                  = 0
SWITCH_CHILD_4m                  = 0
SWITCH_CHILD_5m                  = 0
SWITCH_CHILD_6m                  = 0
SWITCH_CHILD_7m                  = 0
SWITCH_CHILD_8m                  = 0
SWITCH_CHILD_9m                  = 0
SWITCH_CHILD_10m                 = 0
SWITCH_CHILD_11m                 = 0
SWITCH_CHILD_12m                 = 0
SWITCH_CHILD_13m                 = 0
SWITCH_CHILD_14m                 = 0
SWITCH_CHILD_15m                 = 0
SWITCH_CHILD_16m                 = 0
SWITCH_CHILD_17m                 = 0
SWITCH_CHILD_18m                 = 0
SWITCH_CHILD_19m                 = 0
SWITCH_CHILD_20m                 = 0
```

```
SWITCH_CHILD_21m                     = 0
SWITCH_CHILD_22m                     = 0
SWITCH_CHILD_23m                     = 0
SWITCH_CHILD_24m                     = 0
SWITCH_MOTHER_20y                    = 0
SWITCH_CONCEPTION                    = 0
SWITCH_DELIVERY                      = 0
SWITCH_POSTPARTUM_WEIGHT_1           = 0
SWITCH_POSTPARTUM_WEIGHT_2           = 0
SWITCH_BREASTFEEDING                 = 1
SWITCH_END_EXCLUSIVE_BREASTFEEDING = 0
SWITCH_END_TOTAL_BREASTFEEDING       = 0
SWITCH_AGE_CHILD_WEIGHT1             = 0
SWITCH_AGE_CHILD_WEIGHT2             = 0
SWITCH_AGE_CHILD_WEIGHT3             = 0
C_MOTHER_3RULE                       = 0
end


schedule child1m .xn. (0.0833-AGE_child)
discrete child1m
       SWITCH_CHILD_1m = 1
End
schedule child2m .xn. (0.167-AGE_child)
discrete child2m
       SWITCH_CHILD_2m = 1
End
schedule child3m .xn. (0.250-AGE_child)
discrete child3m
       SWITCH_CHILD_3m = 1
End
schedule child4m .xn. (0.333-AGE_child)
discrete child4m
       SWITCH_CHILD_4m = 1
End
schedule child5m .xn. (0.417-AGE_child)
discrete child5m
       SWITCH_CHILD_5m = 1
End
schedule child6m .xn. (0.500-AGE_child)
discrete child6m
       SWITCH_CHILD_6m = 1
End
schedule child7m .xn. (0.583-AGE_child)
discrete child7m
       SWITCH_CHILD_7m = 1
End
```

```
schedule child8m .xn. (0.667-AGE_child)
discrete child8m
       SWITCH_CHILD_8m = 1
End
schedule child9m .xn. (0.750-AGE_child)
discrete child9m
       SWITCH_CHILD_2m = 1
End
schedule child10m .xn. (0.833-AGE_child)
discrete child10m
       SWITCH_CHILD_10m = 1
End
schedule child11m .xn. (0.917-AGE_child)
discrete child11m
       SWITCH_CHILD_11m = 1
End
schedule child12m .xn. (1.000-AGE_child)
discrete child12m
       SWITCH_CHILD_12m = 1
End
schedule child13m .xn. (1.0833-AGE_child)
discrete child13m
       SWITCH_CHILD_13m = 1
End
schedule child14m .xn. (1.167-AGE_child)
discrete child14m
       SWITCH_CHILD_14m = 1
End
schedule child15m .xn. (1.250-AGE_child)
discrete child15m
       SWITCH_CHILD_15m = 1
End
schedule child16m .xn. (1.333-AGE_child)
discrete child16m
       SWITCH_CHILD_16m = 1
End
schedule child17m .xn. (1.417-AGE_child)
discrete child17m
       SWITCH_CHILD_17m = 1
End
schedule child18m .xn. (1.500-AGE_child)
discrete child18m
       SWITCH_CHILD_18m = 1
End
schedule child19m .xn. (1.583-AGE_child)
discrete child19m
       SWITCH_CHILD_19m = 1
```

```
End
schedule child20m .xn. (1.667-AGE_child)
discrete child20m
       SWITCH_CHILD_20m = 1
End
schedule child21m .xn. (1.750-AGE_child)
discrete child21m
       SWITCH_CHILD_21m = 1
End
schedule child22m .xn. (1.833-AGE_child)
discrete child22m
       SWITCH_CHILD_22m = 1
End
schedule child23m .xn. (1.917-AGE_child)
discrete child23m
       SWITCH_CHILD_23m = 1
End
schedule child24m .xn. (2.000-AGE_child)
discrete child24m
       SWITCH_CHILD_24m = 1
End
schedule mother20y .xn. (20-AGE)
discrete mother20y
       SWITCH_MOTHER_20y = 1
End
schedule conception .xn. (AGE_CONCEPTION-AGE)
discrete conception
       SWITCH_CONCEPTION = 1
End
schedule mothersample .xn. (MOTHER_AGE_SAMPLE-AGE)
discrete mothersample
  C_MOTHER_3RULE = C_MOTHER
End
schedule delivery .xn. (AGE_delivery-AGE)
discrete delivery
       SWITCH_DELIVERY = 1
End
schedule agepostpartumweight1 .xn. (AGE_POSTPARTUM_WEIGHT_1-AGE)
discrete agepostpartumweight1
       SWITCH_POSTPARTUM_WEIGHT_1 = 1
End
schedule agepostpartumweight2 .xn. (AGE_POSTPARTUM_WEIGHT_2-AGE)
discrete agepostpartumweight2
       SWITCH_POSTPARTUM_WEIGHT_2 = 1
End
if(DURATION_TOTAL_Breastfeeding .eq. 0) then
  SWITCH_BREASTFEEDING = 0
```

```
endif
schedule endexclbf .xn. (DURATION_EXCL_Breastfeeding-AGE_child)
discrete endexclbf
       SWITCH_END_EXCLUSIVE_BREASTFEEDING = 1
End
schedule endtotalbf .xn. (DURATION_TOTAL_Breastfeeding-AGE_child)
discrete endtotalbf
       SWITCH_END_TOTAL_BREASTFEEDING = 1
End
schedule agechildweight1 .xn. (AGE_CHILD_WEIGHT_1-AGE_child)
discrete agechildweight1
       SWITCH_AGE_CHILD_WEIGHT1 = 1
End
schedule agechildweight2 .xn. (AGE_CHILD_WEIGHT_2-AGE_child)
discrete agechildweight2
       SWITCH_AGE_CHILD_WEIGHT2 = 1
End
schedule agechildweight3 .xn. (AGE_CHILD_WEIGHT_3-AGE_child)
discrete agechildweight3
       SWITCH_AGE_CHILD_WEIGHT3 = 1
End


!*************************************************************************************
! Mass balance differential equations

RA_MOTHER = RAO-ELIMINATION-MOTHER_FETUS_TRANSFER+FETUS_MOTHER_TRANSFER- &
            BREASTMILK_TRANSFER                                              ! Rate of amount in maternal compartment (ug/h)
A_MOTHER  = INTEG(RA_MOTHER, 0.0)                                            ! Amount in maternal compartment (ug)
C_MOTHER  = A_MOTHER/(VOL_FAT_MOTHER)                                        ! Concentration in maternal lipids (ug/kg or ng/g)

RA_CHILD  = MOTHER_FETUS_TRANSFER-FETUS_MOTHER_TRANSFER- &
            ELIMINATION_child+BREASTMILK_TRANSFER                            ! Rate of amount of POPs in child compartment (ug/h)
A_CHILD   = INTEG(RA_CHILD, 0.0)                                             ! Amount in child compartment (ug)
C_CHILD   = A_CHILD/(VOL_FAT_CHILD)                                          ! Concentration in child lipids (ug/kg or ng/g)


!*************************************************************************************
! Maximum blood concentration in child

initial
MAX_C_CHILD = 0
TMAX_C_CHILD = 0
end
if(C_CHILD>MAX_C_CHILD .AND. AGE_child >0 .AND. AGE_child <3.75)then
MAX_C_CHILD=C_CHILD
TMAX_C_CHILD=AGE_child
```

```
Endif

!*****************************************************************************************
! Area under the curve

AUC_CHILD  = INTEG(C_CHILD, 0.0)
AUC_MOTHER = INTEG(C_MOTHER, 0.0)


!*****************************************************************************************
! Save monthly concentrations

initial
C1M  = 0
C2M  = 0
C3M  = 0
C4M  = 0
C5M  = 0
C6M  = 0
C7M  = 0
C8M  = 0
C9M  = 0
C10M = 0
C11M = 0
C12M = 0
C13M = 0
C14M = 0
C15M = 0
C16M = 0
C17M = 0
C18M = 0
C19M = 0
C20M = 0
C21M = 0
C22M = 0
C23M = 0
C24M = 0
C25M = 0
C26M = 0
C27M = 0
C28M = 0
C29M = 0
C30M = 0
C31M = 0
C32M = 0
C33M = 0
C34M = 0
```

```
C35M = 0
C36M = 0
C37M = 0
C38M = 0
C39M = 0
C40M = 0
C41M = 0
C42M = 0
C43M = 0
C44M = 0
C45M = 0
end

schedule c1mrecord .xn. (0.083-AGE_Child)
discrete c1mrecord
C1M = C_CHILD
end
schedule c2mrecord .xn. (0.167-AGE_Child)
discrete c2mrecord
C2M = C_CHILD
end
schedule c3mrecord .xn. (0.250-AGE_Child)
discrete c3mrecord
C3M = C_CHILD
end
schedule c4mrecord .xn. (0.333-AGE_Child)
discrete c4mrecord
C4M = C_CHILD
end
schedule c5mrecord .xn. (0.417-AGE_Child)
discrete c5mrecord
C5M = C_CHILD
end
schedule c6mrecord .xn. (0.500-AGE_Child)
discrete c6mrecord
C6M = C_CHILD
end
schedule c7mrecord .xn. (0.583-AGE_Child)
discrete c7mrecord
C7M = C_CHILD
end
schedule c8mrecord .xn. (0.667-AGE_Child)
discrete c8mrecord
C8M = C_CHILD
end
schedule c9mrecord .xn. (0.750-AGE_Child)
discrete c9mrecord
```

```
C9M = C_CHILD
end
schedule c10mrecord .xn. (0.833-AGE_Child)
discrete c10mrecord
C10M = C_CHILD
end
schedule c11mrecord .xn. (0.917-AGE_Child)
discrete c11mrecord
C11M = C_CHILD
end
schedule c12mrecord .xn. (1.0-AGE_Child)
discrete c12mrecord
C12M = C_CHILD
end
schedule c13mrecord .xn. (1.083-AGE_Child)
discrete c13mrecord
C13M = C_CHILD
end
schedule c14mrecord .xn. (1.167-AGE_Child)
discrete c14mrecord
C14M = C_CHILD
end
schedule c15mrecord .xn. (1.250-AGE_Child)
discrete c15mrecord
C15M = C_CHILD
end
schedule c16mrecord .xn. (1.333-AGE_Child)
discrete c16mrecord
C16M = C_CHILD
end
schedule c17mrecord .xn. (1.417-AGE_Child)
discrete c17mrecord
C17M = C_CHILD
end
schedule c18mrecord .xn. (1.500-AGE_Child)
discrete c18mrecord
C18M = C_CHILD
end
schedule c19mrecord .xn. (1.583-AGE_Child)
discrete c19mrecord
C19M = C_CHILD
end
schedule c20mrecord .xn. (1.667-AGE_Child)
discrete c20mrecord
C20M = C_CHILD
end
schedule c21mrecord .xn. (1.750-AGE_Child)
```

```
discrete c21mrecord
C21M = C_CHILD
end
schedule c22mrecord .xn. (1.833-AGE_Child)
discrete c22mrecord
C22M = C_CHILD
end
schedule c23mrecord .xn. (1.917-AGE_Child)
discrete c23mrecord
C23M = C_CHILD
end
schedule c24mrecord .xn. (2.0-AGE_Child)
discrete c24mrecord
C24M = C_CHILD
end
schedule c25mrecord .xn. (2.083-AGE_Child)
discrete c25mrecord
C25M = C_CHILD
end
schedule c26mrecord .xn. (2.167-AGE_Child)
discrete c26mrecord
C26M = C_CHILD
end
schedule c27mrecord .xn. (2.250-AGE_Child)
discrete c27mrecord
C27M = C_CHILD
end
schedule c28mrecord .xn. (2.333-AGE_Child)
discrete c28mrecord
C28M = C_CHILD
end
schedule c29mrecord .xn. (2.417-AGE_Child)
discrete c29mrecord
C29M = C_CHILD
end
schedule c30mrecord .xn. (2.500-AGE_Child)
discrete c30mrecord
C30M = C_CHILD
end
schedule c31mrecord .xn. (2.583-AGE_Child)
discrete c31mrecord
C31M = C_CHILD
end
schedule c32mrecord .xn. (2.667-AGE_Child)
discrete c32mrecord
C32M = C_CHILD
end
```

```
schedule c33mrecord .xn. (2.750-AGE_Child)
discrete c33mrecord
C33M = C_CHILD
end
schedule c34mrecord .xn. (2.833-AGE_Child)
discrete c34mrecord
C34M = C_CHILD
end
schedule c35mrecord .xn. (2.917-AGE_Child)
discrete c35mrecord
C35M = C_CHILD
end
schedule c36mrecord .xn. (3.0-AGE_Child)
discrete c36mrecord
C36M = C_CHILD
end
schedule c37mrecord .xn. (3.083-AGE_Child)
discrete c37mrecord
C37M = C_CHILD
end
schedule c38mrecord .xn. (3.167-AGE_Child)
discrete c38mrecord
C38M = C_CHILD
end
schedule c39mrecord .xn. (3.250-AGE_Child)
discrete c39mrecord
C39M = C_CHILD
end
schedule c40mrecord .xn. (3.333-AGE_Child)
discrete c40mrecord
C40M = C_CHILD
end
schedule c41mrecord .xn. (3.417-AGE_Child)
discrete c41mrecord
C41M = C_CHILD
end
schedule c42mrecord .xn. (3.500-AGE_Child)
discrete c42mrecord
C42M = C_CHILD
end
schedule c43mrecord .xn. (3.583-AGE_Child)
discrete c43mrecord
C43M = C_CHILD
end
schedule c44mrecord .xn. (3.667-AGE_Child)
discrete c44mrecord
C44M = C_CHILD
```

```
end
schedule c45mrecord .xn. (3.750-AGE_Child)
discrete c45mrecord
C45M = C_CHILD
end


END ! DERIVATIVE

END ! DYNAMIC

END ! PROGRAM
```

M FILE

```
prepare @all
save

CIEITG =0;
WESITG=0;
WXDITG=0;
WEDITG=0;
CJVITG=0;
WNDITG=0;

if(1)
CINT=24
MINT=0.1
MAXT=24.
end

%*********************************************************************************%
% Commands to read from Excel spreadsheet                                         %
% Specify the file path, the sheet to read from and the array                     %
%*********************************************************************************%

Individual                     = xlsread("c:\Dataset_cohort1.xls", "Sheet1", "A2:A500") ;    % Individual number (from 1 to ...)
IDs                            = xlsread("c:\Dataset_cohort1.xls", "Sheet1", "B2:B500") ;    % Study ID
Age_deliverys                  = xlsread("c:\Dataset_cohort1.xls", "Sheet1", "C2:C500") ;    % Age at delivery (years)
Prepgregnancy_bws              = xlsread("c:\Dataset_cohort1.xls", "Sheet1", "D2:D500") ;    % Pre-pregnancy weight (kg)
Weight_gain_pregnancys         = xlsread("c:\Dataset_cohort1.xls", "Sheet1", "E2:E500") ;    % Weight gain end of pregnancy (kg)
Postpartum_weight_1s           = xlsread("c:\Dataset_cohort1.xls", "Sheet1", "F2:F500") ;    % Weight after delivery 1st measure (kg)
Postpartum_weight_2s           = xlsread("c:\Dataset_cohort1.xls", "Sheet1", "G2:G500") ;    % Weight after delivery 2nd measure (kg)
Age_postpartum_weight_1s       = xlsread("c:\Dataset_cohort1.xls", "Sheet1", "H2:H500") ;    % Age 1st postpartum weighing (years)
Age_postpartum_weight_2s       = xlsread("c:\Dataset_cohort1.xls", "Sheet1", "I2:I500") ;    % Age 2nd postpartum weighing (years)
Sexs                           = xlsread("c:\Dataset_cohort1.xls", "Sheet1", "J2:J500") ;    % Child sex (0:female, 1:male)
Gestational_ages               = xlsread("c:\Dataset_cohort1.xls", "Sheet1", "K2:K500") ;    % Gestational age (in years)
Birthweights                   = xlsread("c:\Dataset_cohort1.xls", "Sheet1", "L2:L500") ;    % Birth weight (kg)
Child_weight_1s                = xlsread("c:\Dataset_cohort1.xls", "Sheet1", "M2:M500") ;    % Child weight at first measure (kg)
Child_weight_2s                = xlsread("c:\Dataset_cohort1.xls", "Sheet1", "N2:N500") ;    % Child weight at second measure (kg)
Child_weight_3s                = xlsread("c:\Dataset_cohort1.xls", "Sheet1", "O2:O500") ;    % Child weight at third measure (kg)
Age_child_weight_1s            = xlsread("c:\Dataset_cohort1.xls", "Sheet1", "P2:P500") ;    % Age of child at 1st weighing (years)
Age_child_weight_2s            = xlsread("c:\Dataset_cohort1.xls", "Sheet1", "Q2:Q500") ;    % Age of child at 2nd weighing (years)
Age_child_weight_3s            = xlsread("c:\Dataset_cohort1.xls", "Sheet1", "R2:R500") ;    % Age of child at 3rd weighing (years)
Duration_excl_breastfeedings   = xlsread("c:\Dataset_cohort1.xls", "Sheet1", "S2:S500") ;    % Exclusive breast-feeding (years)
Duration_total_breastfeedings  = xlsread("c:\Dataset_cohort1.xls", "Sheet1", "T2:T500") ;    % Total breast-feeding (years)
Fraction_bf_1ms                = xlsread("c:\Dataset_cohort1.xls", "Sheet1", "U2:U500") ;    % Fraction of exclusive BF (month 1)
Fraction_bf_2ms                = xlsread("c:\Dataset_cohort1.xls", "Sheet1", "V2:V500") ;    % Fraction of exclusive BF (month 2)
Fraction_bf_3ms                = xlsread("c:\Dataset_cohort1.xls", "Sheet1", "W2:W500") ;    % Fraction of exclusive BF (month 3)
```

```
Fraction_bf_4ms              = xlsread("c:\Dataset_cohort1.xls", "Sheet1", "X2:X500") ;    % Fraction of exclusive BF (month 4)
Fraction_bf_5ms              = xlsread("c:\Dataset_cohort1.xls", "Sheet1", "Y2:Y500") ;    % Fraction of exclusive BF (month 5)
Fraction_bf_6ms              = xlsread("c:\Dataset_cohort1.xls", "Sheet1", "Z2:Z500") ;    % Fraction of exclusive BF (month 6)
Fraction_bf_7ms              = xlsread("c:\Dataset_cohort1.xls", "Sheet1", "AA2:AA500") ;  % Fraction of exclusive BF (month 7)
Fraction_bf_8ms              = xlsread("c:\Dataset_cohort1.xls", "Sheet1", "AB2:AB500") ;  % Fraction of exclusive BF (month 8)
Fraction_bf_9ms              = xlsread("c:\Dataset_cohort1.xls", "Sheet1", "AC2:AC500") ;  % Fraction of exclusive BF (month 9)
Fraction_bf_10ms             = xlsread("c:\Dataset_cohort1.xls", "Sheet1", "AD2:AD500") ;  % Fraction of exclusive BF (month 10)
Fraction_bf_11ms             = xlsread("c:\Dataset_cohort1.xls", "Sheet1", "AE2:AE500") ;  % Fraction of exclusive BF (month 11)
Fraction_bf_12ms             = xlsread("c:\Dataset_cohort1.xls", "Sheet1", "AF2:AF500") ;  % Fraction of exclusive BF (month 12)
Fraction_bf_13ms             = xlsread("c:\Dataset_cohort1.xls", "Sheet1", "AG2:AG500") ;  % Fraction of exclusive BF (month 13)
Fraction_bf_14ms             = xlsread("c:\Dataset_cohort1.xls", "Sheet1", "AH2:AH500") ;  % Fraction of exclusive BF (month 14)
Fraction_bf_15ms             = xlsread("c:\Dataset_cohort1.xls", "Sheet1", "AI2:AI500") ;  % Fraction of exclusive BF (month 15)
Fraction_bf_16ms             = xlsread("c:\Dataset_cohort1.xls", "Sheet1", "AJ2:AJ500") ;  % Fraction of exclusive BF (month 16)
Fraction_bf_17ms             = xlsread("c:\Dataset_cohort1.xls", "Sheet1", "AK2:AK500") ;  % Fraction of exclusive BF (month 17)
Fraction_bf_18ms             = xlsread("c:\Dataset_cohort1.xls", "Sheet1", "AL2:AL500") ;  % Fraction of exclusive BF (month 18)
Fraction_bf_19ms             = xlsread("c:\Dataset_cohort1.xls", "Sheet1", "AM2:AM500") ;  % Fraction of exclusive BF (month 19)
Fraction_bf_20ms             = xlsread("c:\Dataset_cohort1.xls", "Sheet1", "AN2:AN500") ;  % Fraction of exclusive BF (month 20)
Fraction_bf_21ms             = xlsread("c:\Dataset_cohort1.xls", "Sheet1", "AO2:AO500") ;  % Fraction of exclusive BF (month 21)
Fraction_bf_22ms             = xlsread("c:\Dataset_cohort1.xls", "Sheet1", "AP2:AP500") ;  % Fraction of exclusive BF (month 22)
Fraction_bf_23ms             = xlsread("c:\Dataset_cohort1.xls", "Sheet1", "AQ2:AQ500") ;  % Fraction of exclusive BF (month 23)
Fraction_bf_24ms             = xlsread("c:\Dataset_cohort1.xls", "Sheet1", "AR2:AR500") ;  % Fraction of exclusive BF (month 24)
Pop_levels                   = xlsread("c:\Dataset_cohort1.xls", "Sheet1", "AS2:AS500") ;  % POP level in maternal blood, cord
blood or milk lipids (ng/g lipids)
Mother_age_samples           = xlsread("c:\Dataset_cohort1.xls", "Sheet1", "AT2:AT500") ;  % Mother's age at blood/milk sample
(years)
Compounds                    = xlsread("c:\Dataset_cohort1.xls", "Sheet1", "AU2:AU500") ;  % Compound 1:PCB52, 2:PCB105, 3:PCB118
                                                                                            % 4:PCB138, 5:PCB153, 6:PCB170,
                                                                                            % 7:PCB180, 8:PCB187, 9:DDE, 10:DDT
                                                                                            % 11:HCB

NumIndividuals=499;         % Indicate the number of individuals

%*****************************************%
% Creates arrays to record simulated data %
%*****************************************%
doses      = [];
Cmax_CHILD = [];
CHILD_1m   = [];
CHILD_2m   = [];
CHILD_3m   = [];
CHILD_4m   = [];
CHILD_5m   = [];
CHILD_6m   = [];
CHILD_7m   = [];
CHILD_8m   = [];
CHILD_9m   = [];
CHILD_10m  = [];
```

```
CHILD_11m  = [];
CHILD_12m  = [];
CHILD_13m  = [];
CHILD_14m  = [];
CHILD_15m  = [];
CHILD_16m  = [];
CHILD_17m  = [];
CHILD_18m  = [];
CHILD_19m  = [];
CHILD_20m  = [];
CHILD_21m  = [];
CHILD_21m  = [];
CHILD_22m  = [];
CHILD_23m  = [];
CHILD_24m  = [];
CHILD_25m  = [];
CHILD_26m  = [];
CHILD_27m  = [];
CHILD_28m  = [];
CHILD_29m  = [];
CHILD_30m  = [];
CHILD_31m  = [];
CHILD_31m  = [];
CHILD_32m  = [];
CHILD_33m  = [];
CHILD_34m  = [];
CHILD_35m  = [];
CHILD_36m  = [];
CHILD_37m  = [];
CHILD_38m  = [];
CHILD_39m  = [];
CHILD_40m  = [];
CHILD_41m  = [];
CHILD_41m  = [];
CHILD_42m  = [];
CHILD_43m  = [];
CHILD_44m  = [];
CHILD_45m  = [];
calculated_c = [];


%********************************%
% Start of automation script     %
%********************************%

for individual=[1:1:NumIndividuals]
      disp(sprintf("Individual #%d of %d", individual, NumIndividuals));
```

```
      disp("----------------------------");

ID                            = IDs(individual);
AGE_DELIVERY                  = Age_deliverys(individual);
PREPREGNANCY_BW               = Prepgregnancy_bws(individual);
WEIGHT_GAIN_PREGNANCY         = Weight_gain_pregnancys(individual);
POSTPARTUM_WEIGHT_1           = Postpartum_weight_1s(individual);
POSTPARTUM_WEIGHT_2           = Postpartum_weight_2s(individual);
AGE_POSTPARTUM_WEIGHT_1       = Age_postpartum_weight_1s(individual);
AGE_POSTPARTUM_WEIGHT_2       = Age_postpartum_weight_2s(individual);
SEX                           = Sexs(individual) ;
GESTATIONAL_AGE               = Gestational_ages(individual);
BIRTHWEIGHT                   = Birthweights(individual);
CHILD_WEIGHT_1                = Child_weight_1s(individual);
CHILD_WEIGHT_2                = Child_weight_2s(individual);
CHILD_WEIGHT_3                = Child_weight_3s(individual);
AGE_CHILD_WEIGHT_1            = Age_child_weight_1s(individual) ;
AGE_CHILD_WEIGHT_2            = Age_child_weight_2s(individual);
AGE_CHILD_WEIGHT_3            = Age_child_weight_3s(individual);
DURATION_EXCL_BREASTFEEDING   = Duration_excl_breastfeedings(individual);
DURATION_TOTAL_BREASTFEEDING = Duration_total_breastfeedings(individual);
FRACTION_BF_1M                = Fraction_bf_1ms(individual);
FRACTION_BF_2M                = Fraction_bf_2ms(individual);
FRACTION_BF_3M                = Fraction_bf_3ms(individual);
FRACTION_BF_4M                = Fraction_bf_4ms(individual);
FRACTION_BF_5M                = Fraction_bf_5ms(individual);
FRACTION_BF_6M                = Fraction_bf_6ms(individual);
FRACTION_BF_7M                = Fraction_bf_7ms(individual);
FRACTION_BF_8M                = Fraction_bf_8ms(individual);
FRACTION_BF_9M                = Fraction_bf_9ms(individual);
FRACTION_BF_10M               = Fraction_bf_10ms(individual);
FRACTION_BF_11M               = Fraction_bf_11ms(individual);
FRACTION_BF_12M               = Fraction_bf_12ms(individual);
FRACTION_BF_13M               = Fraction_bf_13ms(individual);
FRACTION_BF_14M               = Fraction_bf_14ms(individual);
FRACTION_BF_15M               = Fraction_bf_15ms(individual);
FRACTION_BF_16M               = Fraction_bf_16ms(individual);
FRACTION_BF_17M               = Fraction_bf_17ms(individual);
FRACTION_BF_18M               = Fraction_bf_18ms(individual);
FRACTION_BF_19M               = Fraction_bf_19ms(individual);
FRACTION_BF_20M               = Fraction_bf_20ms(individual);
FRACTION_BF_21M               = Fraction_bf_21ms(individual);
FRACTION_BF_22M               = Fraction_bf_22ms(individual);
FRACTION_BF_23M               = Fraction_bf_23ms(individual);
FRACTION_BF_24M               = Fraction_bf_24ms(individual);
POP_LEVEL                     = Pop_levels(individual);
MOTHER_AGE_SAMPLE             = Mother_age_samples(individual);
```

```
COMPOUND                     = Compounds(individual);
DOSE                         = 0.01;

start @nocallback

rule3 = C_MOTHER_3RULE;
DOSE  = POP_LEVEL*0.01/rule3;
doses = [doses; DOSE];

start @nocallback

doses       = DOSE;
Cmax_CHILD  = MAX_C_CHILD;
CHILD_1m = C1M;
CHILD_2m = C2M;
CHILD_3m = C3M;
CHILD_4m = C4M;
CHILD_5m = C5M;
CHILD_6m = C6M;
CHILD_7m = C7M;
CHILD_8m = C8M;
CHILD_9m = C9M;
CHILD_10m = C10M;
CHILD_11m = C11M;
CHILD_12m = C12M;
CHILD_13m = C13M;
CHILD_14m = C14M;
CHILD_15m = C15M;
CHILD_16m = C16M;
CHILD_17m = C17M;
CHILD_18m = C18M;
CHILD_19m = C19M;
CHILD_20m = C20M;
CHILD_21m = C21M;
CHILD_22m = C22M;
CHILD_23m = C23M;
CHILD_24m = C24M;
CHILD_25m = C25M;
CHILD_26m = C26M;
CHILD_27m = C27M;
CHILD_28m = C28M;
CHILD_29m = C29M;
CHILD_30m = C30M;
CHILD_31m = C31M;
CHILD_32m = C32M;
CHILD_33m = C33M;
CHILD_34m = C34M;
```

```
CHILD_35m = C35M;
CHILD_36m = C36M;
CHILD_37m = C37M;
CHILD_38m = C38M;
CHILD_39m = C39M;
CHILD_40m = C40M;
CHILD_41m = C41M;
CHILD_42m = C42M;
CHILD_43m = C43M;
CHILD_44m = C44M;
CHILD_45m = C45M;

calculated_c = [calculated_c;  ID CHILD_1m CHILD_2m CHILD_3m CHILD_4m CHILD_5m CHILD_6m CHILD_7m CHILD_8m CHILD_9m CHILD_10m ...
                                  CHILD_11m CHILD_12m CHILD_13m CHILD_14m CHILD_15m CHILD_16m CHILD_17m CHILD_18m CHILD_19m ...
                                  CHILD_20m CHILD_21m CHILD_22m CHILD_23m CHILD_24m CHILD_25m CHILD_26m CHILD_27m CHILD_28m ...
                                  CHILD_29m CHILD_30m CHILD_31m CHILD_32m CHILD_33m CHILD_34m CHILD_35m CHILD_36m CHILD_37m ...
                                  CHILD_38m CHILD_39m CHILD_40m CHILD_41m CHILD_42m CHILD_43m CHILD_44m CHILD_45m Cmax_CHILD ];
end %individuals

%*********************************************************************************%
% Commands to write results in Excel spreadsheet
% Specify the file path, the sheet to read from and the array
%*********************************************************************************%

xlsWrite("C:\Results_cohort1.xls","Sheet1","A2:AU500",calculated_c)
```
